# Supplementary material for: Hybrid Nanocomposites Based on Poly(2,5-dichloro-3,6-bis(phenylamino)-p-benzoquinone) and MWCNTs: Synthesis, Structure, and the Role of ZnO
Source: Polymers (Basel). 2026 Mar 19;18(6):754. doi: 10.3390/polym18060754 (PMC13030130; doi:10.3390/polym18060754)
Supplement: Supplementary file 1 [file polymers-18-00754-s001.zip › polymers-4190518-supplementary.pdf]

3/19/2026 3:33:06 PM

Compare Results

Old File:		New File:
**polymers-4190518-fc.pdf**	versus	**polymers-4190518.pdf**
**36 pages (3.87 MB)**		**34 pages (5.87 MB)**
3/19/2026 3:31:13 PM		3/19/2026 3:30:45 PM

Total Changes

2331

Content

339 Replacements

107 Insertions

114 Deletions

Styling and Annotations

1370 Styling

401 Annotations

Go to First Change (page 1)

polymers

MDPI

Article

Hybrid Nanocomposites Based on Poly(2,5-dichloro-3,6-bis(phenylamino)-p-benzoquinone) and MWCNTs: Synthesis, Structure, and the Role of ZnO

Svetlana G. Kiseleva *, Galina N. Bondarenko, Dmitriy G. Muratov, Vladimir V. Kozlov, Andrey A. Vasilev and Galina P. Karpacheva

A.V. Topchiev Institute of Petrochemical Synthesis, Russian Academy of Sciences, Leninsky Pr., 29, 119991 Moscow, Russia; bond@ips.ac.ru (G.N.B.); muratov@ips.ac.ru (D.G.M.); kozlov@ips.ac.ru (V.V.K.); raver.vasiljev@mail.ru (A.A.V.); gpk@ips.ac.ru (G.P.K.)

* Correspondence: skisel@ips.ac.ru

Abstract

For the first time, hybrid nanocomposites based on poly(2,5-dichloro-3,6-bis(phenylamino)-p-benzoquinone) (PCPAB) and multi-walled carbon nanotubes (MWCNTs) were obtained and the influence of the preparation method on their structure and functional properties was demonstrated. The nanocomposites were obtained both by ultrasonic mixing of PCPAB and MWCNTs, and via in situ oxidative polymerization of CPAB in the presence of MWCNTs or MWCNTs with the addition of ZnO. The formation of hybrid nanocomposites occurs due to non-covalent interaction (π-stacking) between the graphene structures of the MWCNT surface and the phenyl rings of PCPAB. It was found that during the in situ oxidative polymerization of CPAB in the presence of MWCNTs, the growth of polymer chains occurred in close proximity to the filler surface, which led to the formation of a polymer coating. ZnO particles, localized on MWCNTs, on the one hand, prevent their aggregation, and on the other hand, create additional polymerization reaction centers due to the coordination of the Zn-O bond at the H and O atoms of the monomer. An increase in the concentration of reaction centers as a result led to a 2–2.5-fold reduction in the induction polymerization period. According to SEM data, in this case, a more ordered and denser polymer layer is formed due to intermolecular complexation between the main and side chains of the growing polymer with the participation of Zn^{2+} ions formed as a result of the transformation of ZnO to $ZnCl_2$ in the acidic reaction medium of polymerization. The results of the study of the frequency dependences of conductivity indicate a hopping mechanism of conductivity of nanocomposites. The electrical conductivity of nanocomposites depends on their production method and the MWCNT content and varies between 0.5 and 1.1 $S{\cdot}cm^{-1}$, which is 6–12 times higher than the conductivity of the original polymer. Thermogravimetric analysis revealed that the nanocomposites exhibit enhanced thermal stability compared to PCPAB. The best results were shown by nanocomposites with a higher content of MWCNTs, for which the residual mass at 450 °C was 51–53%.

Keywords: oxidative polymerization; diarylaminodichlorobenzoquinones; multi-walled carbon nanotubes

Check for updates

Academic Editors: Hao Li and Delong He

Received: 20 February 2026
Revised: 13 March 2026
Accepted: 16 March 2026
Published: 19 March 2026

Copyright: © 2026 by the authors. Licensee MDPI, Basel, Switzerland. This article is an open access article distributed under the terms and conditions of the Creative Commons Attribution (CC BY) license.

1. Introduction

To the present day, the development of increasingly advanced materials with a range of properties that meet the demands of modern science and technology remains a highly

relevant topic. Consequently, there is sustained interest in the creation of next-generation composites based on polymers with a conjugated system (PCS) and carbon nanotubes (CNTs) [1–4]. PCS have a unique structure stemming from the distribution of π-electrons along the conjugated chain. Introducing CNTs into the polymer matrix allows for the creation of functional materials characterized by a combination of the physico-chemical properties of both PCS and CNTs [4–14]. The similarity of the chemical structures of CNTs and PCS enhances the synergistic effect of combining the individual parameters of the composite components [15,16]. All this ensures a high potential for the practical application of such composites in the creation of electrode materials for electrochemical power sources [17], supercapacitors [18], batteries [19], sensors [3,5], components for solar cells [20], anti-corrosion coatings [21], membranes [22] and others [4,23].

PANI is often used as a polymer component due to the ease of synthesis, relative low cost, easy doping–dedoping processes, high electrochemical activity, electrical and thermal conductivity. Introducing substituents in the N- and C-positions allows for a significant expansion of the range of polyaniline polymers [24–28], while their structure, physicochemical, and electronic properties can vary considerably depending on the synthesis method (electrochemical, bulk, emulsion, etc.), synthesis conditions, and the type of dopant [29–33]. Doping of PANI with metal ions, in particular (Zn^{2+}), leads to an increased charge delocalization in the polymer chain and, consequently, to an increase in conductivity and electrochemical capacitance [34–36].

Carbon nanotubes are cylindrical structures composed of carbon atoms forming a hexagonal lattice, similar to graphene, and exhibiting high electrical conductivity, high thermal stability, corrosion resistance, a percolated pore structure, and a large surface area [37,38]. CNTs used as a scaffold for the synthesis of composite materials with PANI also make it possible to mitigate the dependence of the electrical conductivity of the composites on the degree of oxidation and protonation of PANI.

The development of methods for obtaining PANI/CNT composite materials takes into consideration the requirements for the shape and type of the final product, defined by its intended area of subsequent application. Research efforts are aimed at obtaining nanocomposites characterized by a finely dispersed distribution of MWCNTs, prone to aggregation, in a polymer matrix. There are several main production routes: mechanical mixing of pre-obtained polymer and CNTs in the solid and liquid phases, chemical in situ polymerization of aniline in the presence of CNTs, and electrochemical polymerization of the monomer in the presence of a carbon filler [39]. The electrochemical method is used for obtaining thin-film coatings and can be applied, for example, to produce electrodes [6,21,40–42]. Direct mixing of components is the simplest method to obtain nanocomposite materials [9,15,43–45]. The disadvantage is the difficulty of ensuring uniform distribution of components in the volume of the nanocomposite [46,47]. Achieving good dispersion of aggregation-prone carbon nanotubes is possible via in situ oxidative polymerization of monomers in the presence of CNTs [15,21,48–52]. On CNTs, the polymer forms a coaxial shell due to π-stacking between the quinodiimine units of PANI and the hexagonal cycles of the graphene surface of the carbon filler, which enhances electron transport in the nanomaterial compared to the pure polymer, thereby improving the conductivity parameters of the final product [16,48,53–56]. The use of ultrasound in the polymerization process further ensures a finer distribution of the carbon filler and prevents its aggregation [57–60].

Despite the undeniable advantages of CNTs, their hydrophobicity, tendency to coagulate and, consequently, insufficient specific surface area and specific capacitance, non-uniform pore distribution, as well as an insufficient number of active groups on the surface, compared, for example, to graphene oxide (GO), make it difficult to achieve the required parameters, and stability of composite materials based on them [61]. To overcome these

shortcomings, methods for covalent functionalization and non-covalent modification of CNTs have been developed [62].

The most common methods of covalent functionalization are: chemical oxidation—treatment with oxygen-containing strong acids and/or their mixtures with oxidizing agents [15,62–69]; gas-phase treatment—using CO_2, water vapor or atmospheric oxygen [70–72]; mechanochemical treatment—treatment of CNTs in aqueous-alcoholic solutions of KOH [73]. As a result, oxygen-containing groups (-COOH, -OH, etc.) are formed on the CNT surface. This improves the affinity of the nanotube surface to various solvents and polymer matrices and facilitates their subsequent use as precursors for secondary functionalization of CNTs: amidation/amination, esterification, grafting of polymer macromolecules, and PCS, among others [62,74–76]. However, this method is multi-stage and requires the use of expensive and often highly toxic reagents [77–79], while high temperatures and aggressive media during processing lead to structural defects in CNTs, which degrades their mechanical, thermal, and electrical properties. In the most recent times, there have been reports on the incorporation of ZnO nanoparticles into nanocomposites based on PANI and functionalized CNTs to enhance their photovoltaic and photocatalytic properties [80,81]. ZnO is characterized by unique catalytic, electrical, and optical properties, as well as a lack of toxicity and low cost [82,83]. ZnO-containing PCS/CNT nanocomposites have also been reported to improve the mechanical and anticorrosion properties and stability of epoxy coatings [84,85].

Non-covalent modification is based on the physical interaction between CNTs and chemical substances without changing the sp^2 hybridization of carbon atoms and the π-π system of filler layers. This is one of the key advantages of this method, as it allows preserving physicochemical characteristics of CNTs [62,86,87]. The Van der Waals forces and π-stacking between CNTs and polymer chains containing aromatic rings ensure the formation of a polymer in close proximity to the CNT surface, which often results in wrapping of polymer chains around tubes and forming supramolecular structures, thereby improving the distribution of the filler in the composite [21,87–98].

Despite a large body of publications dedicated to nanocomposites of conjugated polymers (predominantly PANI) and MWCNTs, the scientific literature lacks information on nanocomposites that include, along with MWCNTs, N-substituted polyanilines with functional groups in the side substituents that enhance the interaction of the polymer with the MWCNT surface. Systematic comparative studies of the composition, chemical structure, and properties of nanocomposites obtained by different methods and differing in the content of carbon filler nanoparticles are lacking. Furthermore, there is no information on the effect of metal oxide additives in nanocomposites on the formation process, chemical structure, and morphology of the polymer layer formed on the surface of non-functionalized MWCNTs.

Previously, we synthesized a novel polymer from the class of N-substituted polyanilines—poly(2,5-dichloro-3,6-bis(phenylamino)-p-benzoquinone) (PCPAB) with has bulky electroactive side substituents containing functional phenylamine groups, and studied the kinetics of the polymerization process, the mechanism of formation of active polymerization centers, the chemical structure and electrical properties of the polymer [99,100]. PCPAB-based nanocomposites, obtained via oxidative polymerization of the monomer in the presence of GO, showed high and stable electrochemical characteristics in an aprotic electrolyte [101]. A significant influence of the preparation conditions on the structure and functional properties of PCPAB nanocomposites with GO was shown [102].

This paper presents the results of the study of the chemical structure, morphology, thermal stability, and electrophysical properties of nanocomposites based on PCPAB and non-functionalized MWCNTs (hereinafter referred to as MWCNTs) on the conditions of their preparation. Nanocomposites were obtained by ultrasonic mixing of PCPAB and

MWCNTs; via in situ oxidative polymerization of CPAB: in the presence of MWCNTs or in the presence of MWCNTs and ZnO powder (MWCNTs/ZnO). ZnO, adsorbed on the surface of MWCNTs, which are excellent adsorbents [103,104], prevents the aggregation of nanotubes. By coordinating with the monomer, ZnO increased the concentration of active polymerization sites in the immediate vicinity of MWCNTs. In an acidic reaction solution, ZnO is transformed into $ZnCl_2$. It was shown for the first time that the resulting Zn^{2+} ions participate in the formation of the structure and morphology of the polymer coating on the surface of MWCNTs, interacting with the functional groups of not only the main chains, but also the side chains of the polymer. The resulting hybrid nanocomposites were characterized by FTIR and Raman spectroscopy, scanning electron microscopy (SEM), and X-ray diffraction analysis (XRD). An increase in thermal stability and electrical conductivity of the obtained nanocomposites was shown compared to PCPAB.

2. Materials and Methods

2.1. Materials

Chloranil (reagent grade) was recrystallized from 1,4-dioxane. Dioxane (reagent-grade) was distilled at 101 °C. Aniline (reagent-grade) was distilled twice at the residual pressure of 1.33 kPa and the temperature of 50 °C. Ammonium peroxydisulfate (reagent-grade) was recrystallized from water at 40 °C. Distilled water was distilled twice. Solutions of HCl (analytical-grade) and NH_4OH (extra-pure-grade) were used without additional purification, ZnO (pure for analysis).

2.2. Synthesis of Composites Based on PCPAB and MWCNTs

Three different methods were developed to obtain composites based on PCPAB and MWCNTs: ultrasonic mixing of PCPAB and MWCNTs in an aqueous HCl solution; in situ oxidative polymerization of CPAB in the presence of MWCNTs; in situ oxidative polymerization of CPAB in the presence of MWCNTs and ZnO.

An MEF 91.1 ultrasonic disperser (MELFIZ-ultrasound, Moscow, Russia) was used to homogenize aqueous suspensions of the components during their preliminary preparation and the preparation of P/MWCNT-1. The operating frequency was 22 kHz, and the maximum ultrasonic intensity was 200–250 $W \cdot cm^{-2}$.

A Biosan MSH-300 magnetic stirrer (BIOSAN, Riga, Latvia) was used to stir the aqueous suspensions of the components during the preparation of PCPAB, PCPAB/ZnO, and P/MWCNT-2.1,2.2,3.1,3.2 samples. The stirring speed was set at ~2100 rpm.

2.2.1. Synthesis of PCPAB and PCPAB/ZnO

The PCPAB polymer was obtained via oxidative polymerization of CPAB in an aqueous acid solution with $(NH_4)_2S_2O_8$ (ammonium peroxydisulfate—APS) used as the oxidant [99,100]. For the preparation of PCPAB with added ZnO, zinc oxide was introduced at the stage of the preparation of an aqueous suspension of the CPAB monomer under ultrasonic dispersion. The polymerization reaction was carried out under the same conditions.

The polymer obtained under these conditions was labeled as PCPAB/ZnO.

2.2.2. Synthesis of PCPAB/MWCNTs by Mixing PCPAB and MWCNTs

Our studies [99,100] reported the preparation of PCPAB by oxidative polymerization of CPAB in an aqueous hydrochloric acid solution using APS as an oxidizing agent. A total of 0.1 g of PCPAB in salt form and 12 wt.% of MWCNTs based on the PCPAB mass were placed in a vessel containing 15 mL of 0.05 M HCl and dispersed using an ultrasonic disperser for 45 min at a temperature of ~5 °C. They were then filtered with a Schott filter, and the precipitate was vacuum-dried. The final product yield was 91%.

The resulting sample was designated as P/MWCNT-1 (Method 1).

2.2.3. Synthesis of PCPAB/MWCNTs via In Situ Oxidative Polymerization of CPAB in the Presence of MWCNTs

The monomer—(2,5-dichloro-3,6-bis(phenylamino)-p-benzoquinone) (CPAB)—was obtained via alkylation reaction of aniline with chloranil following our previously described method [99,100]. The preparation of the reaction mixture for obtaining the composite was carried out in several stages:

- Separately, using ultrasonic dispersion under cooling (~5 °C), 2 suspensions were prepared: (1) 0.002 mol of the CPAB monomer were gradually added into 30 mL of water, and the suspension was sonicated for 15 min; and (2) MWCNTs weight portion (6 (12) wt% of CPAB) was added to 20 mL of water, and the suspension was sonicated for 10 min.
- A suspension of CPAB was added to the vessel with the MWCNTs suspension and additionally dispersed for 15 min and dried at 60 °C for 24 h.
- While stirring, 10 mL of HCl solution of a certain concentration was added to the resulting mixture (to obtain a reaction solution of the required concentration) and thermostatted at 18 °C.

To initiate the polymerization, 0.0025 mol of an oxidizing agent—APS—were added instantaneously to 10 mL of HCl solution. The process continued for 4 h (T = 18 °C). The product was filtered with a Schott filter. The prepared composite was washed (0.2 M HCl) and dried in a vacuum dryer until constant weight. The yield was 82–86%.

Samples with a MWCNT content of 6 wt.% were designated as P/MWCNT-2.1, and those with a content of 12 wt.% were designated as P/MWCNT-2.2. (Method 2).

2.2.4. Synthesis of P CPAB/MWCNTs via In Situ Oxidative Polymerization of CPAB in the Presence of MWCNTs and ZnO

The reaction mixture for obtaining the composite was prepared in several stages:

- Separately, using ultrasonic dispersion under cooling (~5 °C), 2 suspensions were prepared: (1) MWCNT weight portion (6 (12) wt% of CPAB) and ZnO 25 wt% of CPAB were added to 30 mL of water, and the suspension was sonicated for 15 min; and (2) 0.002 mol of the CPAB monomer was added to 20 mL of water, and the suspension was sonicated for 10 min.
- A suspension of CPAB was added to the vessel with the MWCNT and ZnO (MWCNT/ZnO) suspension and additionally dispersed for 15 min.
- While stirring, 10 mL of HCl solution of a certain concentration was added to the resulting mixture (to obtain a reaction solution of the required concentration) and thermostatted at 18 °C.

To initiate the polymerization, 0.0025 mol of the oxidant (APS) were added instantaneously to 10 mL of HCl solution. The process continued for 4 h (T = 18 °C). The product was filtered with a Schott filter. The prepared composite was washed (0.2 M HCl) and dried in a vacuum dryer until constant weight. The yield was 85–89%.

Samples with a MWCNTs content of 6 wt.% were designated as P/MWCNT-3.1, and those with a content of 12 wt.% were designated as P/MWCNT-3.2 (Method 3).

Thus, the nanocomposite synthesis methodology has been developed for the first time specifically for the new PCPAB polymer. In contrast to the complex methodology of growing ZnO crystals on the surface of CNTs, we have developed a new and simple methodology for obtaining a joint suspension of CNTs and ZnO powder as a reaction medium for the oxidative polymerization of CPAB.

https://doi.org/10.3390/polym18060000

2.3. Materials Characterization

Fourier transform infrared (FTIR) spectra were recorded in the ATR mode using a HYPERION-2000 IR microscope coupled with a Bruker IFS 66 V/s FTIR spectrometer (Ge crystal, scan 100, resolution 2 cm^{-1}, range 600–4000 cm^{-1}) (Bruker Optik GmbH, Ettlingen, Germany). Optical density was D = lgI_0/I.

Powder X-ray diffraction (XRD) analysis was performed using a diffractometer "Difray" 401 (Scientific Instruments, Moscow, Russia) with Bragg–Brentano focusing, using Cr-Kα (wavelength 0.22909 nm) radiations.

Thermal properties of the composites were analyzed by thermogravimetric analysis (TGA) using a TA Instruments Q500 analyzer (TA Instruments, New Castle, DE, USA). TGA curves were obtained under a nitrogen atmosphere (40 mL/min) and a heating rate of 10 °C/min from room temperature to 800 °C. The derivative thermogravimetric (DTG) curves were directly derived from the original TGA thermogram using the product software.

Thermal properties of the composites were analyzed by thermogravimetric analysis (TGA) using a TA Instruments Discovery TG TM unit (TA Instruments, New Castle, DE, USA). TGA curves were obtained under a nitrogen atmosphere (10 mL/min) and a heating rate of 15 °C/min in the temperature range of 30–450 °C. The mass of the sample was 2–4 mg. The derived thermogravimetric (DTG) curves were obtained directly from the original TGA thermogram using the product software (TRIOS software, V4.3, 2012).

FE-SEM images were taken using a Zeiss Supra 25 FE-SEM field emission scanning electron microscope (Carl Zeiss AG, Jena, Germany). Image resolution is 1–2 nm.

Raman spectra were recorded on a Senterra II Raman spectrometer (Bruker, Karlsruhe, Germany) using a laser with a wavelength of 532 nm and a power of 0.25 mW, with spectral resolution of 4 cm^{-1}.

Kinetic studies of the oxidative polymerization of CPAB were carried out by the potentiometric method using a 4-channel ion meter "Expert-001–3(0.4)" ("Econix-Expert", Moscow, Russia) with the accuracy of EMF = ±1.5 mV, recording changes in EMF in correlation with the reaction time. A redoxmetric electrode ERP-105 (Russia) was used as an electrode. As part of the kinetic studies, the polymerization conditions had the following parameters: [CPAB] = 0.03 mol/L; [APS]/[CPAB] = 1.25; [HCl] = 0.5 mol/L; T = 18 °C. The amount of GO was calculated in weight percentage of CPAB.

The AC conductivity was measured with E7-20 precision LCR meter (OJSC "MNIPI", Minsk, Rep. Belarus) in the frequency range of 25 Hz–1.0 MHz. Impedance measurements (frequency dependences of electrical conductivity) using a two-electrode circuit, on alternating current of a given frequency. The sample diameter is 6 mm. The thickness is 3–4 mm. The measurement temperature is constant (23 °C). Measurements for each sample were taken three times and averaged. The frequency dependences of electrical conductivity were approximated using the OriginLab software package (OriginPro 2018 (b9.5.1.195)).

Zn content was determined using inductively coupled plasma atomic emission spectroscopy (ICP-AES) (Shimadzu, Kyoto, Japan).

3. Results and Discussion

3.1. Structure of PCPAB/MWCNT Composites

Nanocomposites were obtained in three different ways: (1) ultrasonic mixing of previously prepared PCPAB and MWCNTs (P/MWCNT-1); in situ oxidative polymerization of CPAB; (2) in the presence of MWCNTs (P/MWCNT-2); (3) in the presence of MWCNT/ZnO (P/MWCNT-3) (Scheme 1).

https://doi.org/10.3390/polym18060000

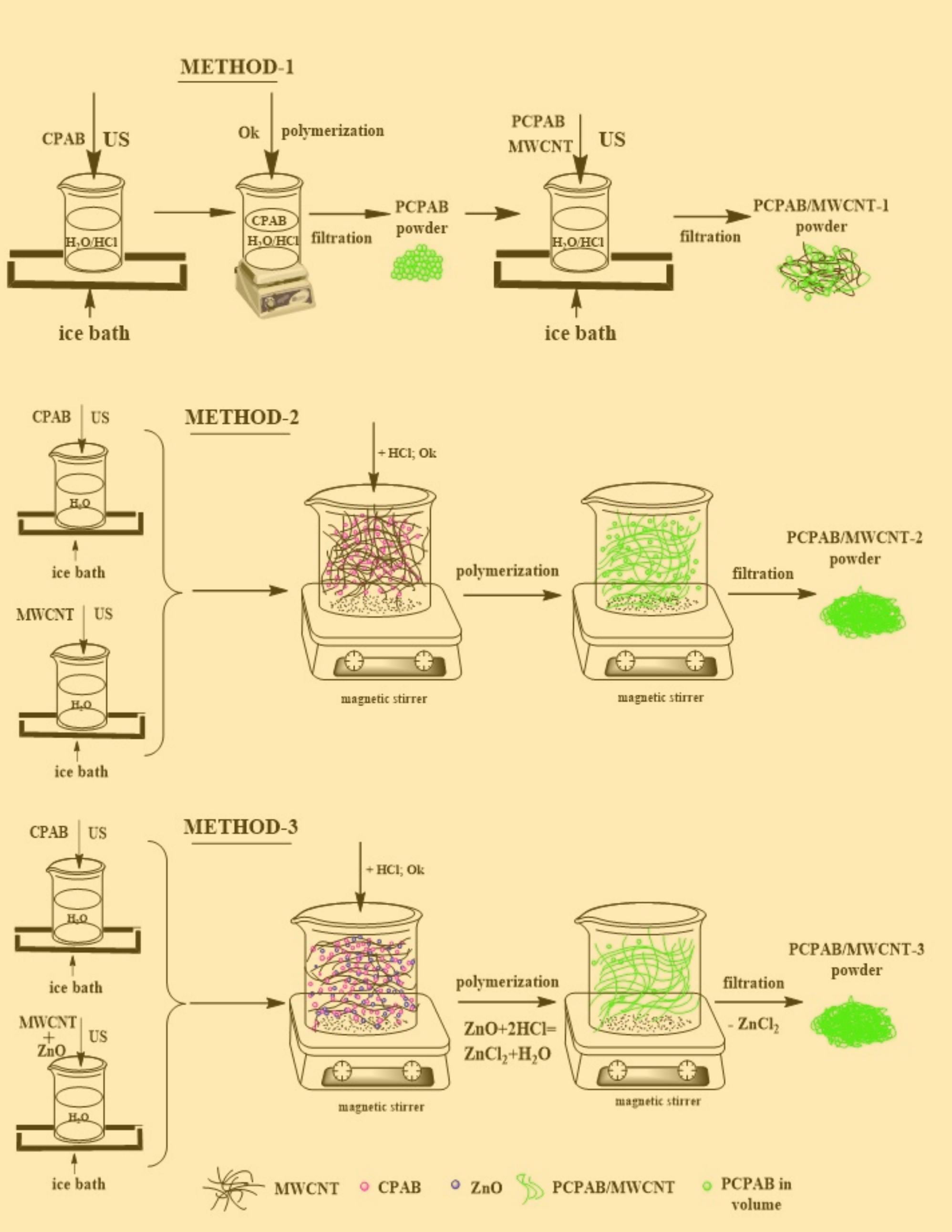

Scheme 1. A schematic view of the synthesis process of a composite.

At the suspension preparation stage, water-insoluble zinc oxide was dispersed with MWCNTs using ultrasound, distributing it between the tubes and over their surface. This ensured a more uniform distribution of MWCNTs in the aqueous medium, as well as the formation of additional polymerization reaction centers concentrated near the MWCNTs. This results in a uniform polymer coating on the MWCNT surface. Only after the monomer has been introduced into the aqueous MWCNT/ZnO suspension and treated with ultrasound is the mixture adjusted to the required pH by adding the necessary amount of acid. It should be noted that during polymerization in an acidic reaction medium, ZnO gradually transforms into $ZnCl_2$ salt and water and, as shown in Table 1, is almost completely removed during composite isolation process (Table 1). The amount of residual Zn in the P/MWCNT-3.1 and P/MWCNT-3.2 composites is determined by the MWCNTs content.

https://doi.org/10.3390/polym18060000

Table 1. Metal content in the samples according to ICP-AES analysis data.

Sample	wt%
PCPAB/ZnO	<detection limit
P/MWCNT-3.1	0.1
P/MWCNT-3.2	0.4

In this study, PCPAB in its conductive salt form was used as the polymer component (Scheme 2).

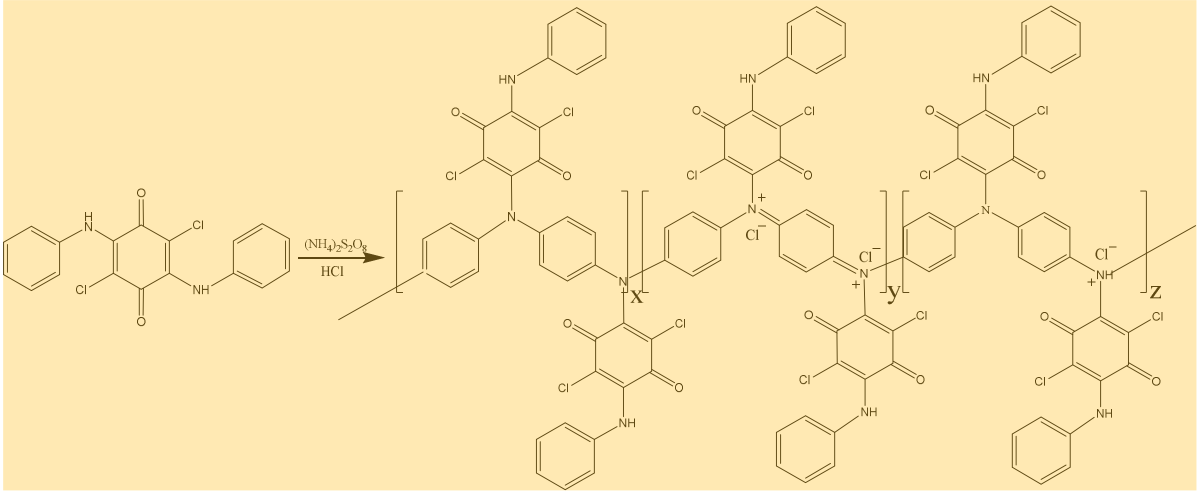

Scheme 2. The structure of PCPAB as cast in salt form.

In the spectrum of PCPAB in its salt form (Figure 1, spectrum 1), the region of absorption bands of N-H bonds demonstrates a narrow band at 3232 cm^{-1} associated with $\nu_{N\text{-}H}$ stretching vibrations in the diphenylamine groups in the PCPAB structure, and a broad split band at 2800–2900 cm^{-1} associated with the N-H bonds in the ammonium cations in the salt form of the polymer. The structure of PCPAB in the IR spectra is characterized by bands associated with the stretching vibrations of C=C bonds in aromatic rings (1600–1480 cm^{-1}), δCCH deformation vibrations in monosubstituted aromatic rings (693 and 750 cm^{-1}) and at 818 cm^{-1} in para-disubstituted aromatic rings, as well as bands at 1245 and 1306 cm^{-1} associated with N-C bonds. Quinone rings in the polymer structure were characterized by bands of C=O stretching vibrations (1650 cm^{-1} and 1600 cm^{-1}) and of ring deformation vibrations (909 and 712 cm^{-1}). Deformation vibrations in the ammonium cations present in the salt form of the polymer (>N^+=Qu; -NH^+-B) were characterized by a band of medium intensity at 801 cm^{-1} and a broad band of high intensity in the region of 1141 cm^{-1} [102]. To show the changes in the structure of PCPAB obtained upon the addition of ZnO to the reaction mixture, the IR spectrum of PCPAB/ZnO (spectrum 2) is presented in Figure 1. A significant reduction in the quantity of ammonium cations is observed, as evidenced by a decrease in the intensity of the characteristic band at 1141 cm^{-1} and a shift to higher frequencies, which indicates a decrease in the quantity of cations bound to the polymer. Furthermore, two overlapping bands—at 820 cm^{-1} associated with out-of-plane deformation vibrations in 1,4-substituted aromatic rings of the polymer and at 803 cm^{-1} associated with deformation vibrations of ammonium cations—are shifted to higher frequencies, and the intensity of the I_{803} band is lower than that of the I_{820} band, unlike in the spectrum of PCPAB. This indicates a decrease in the degree of doping, which is associated with a drop in the pH of the reaction mixture due to the dissolution of zinc oxide with the formation of salt and water. The changes in the structure of PCPAB/ZnO

are confirmed by XRD data. The diffraction pattern of PCPAB (1, Figure 2) features a series of peaks of varying intensity in the range of 18.4° < 2θ < 48°. These peaks are attributed to the crystalline phase in the polymer [102,105,106]. The peaks in the diffraction pattern of PCPAB/ZnO (2, Figure 2) became broader and more split, and their intensity decreased. This is due to a decrease in the degree of doping and, consequently, a decrease in the degree of crystallinity of the polymer obtained with ZnO in the bulk of the reaction solution [107–111].

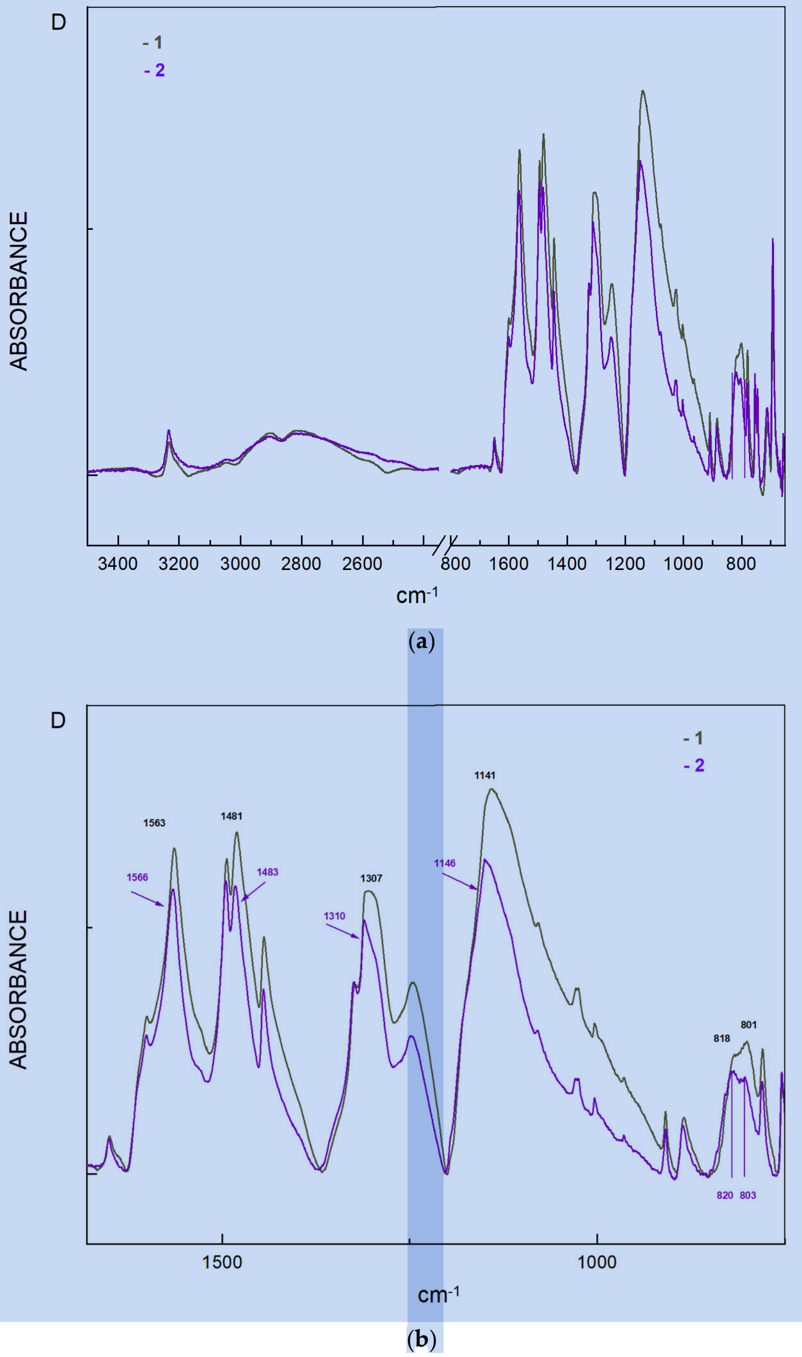

Figure 1. (**a**) Comparison of IR spectra of PCPAB (1); PCPAB/ZnO (2) in salt form; (**b**) detailing of the spectrum in the region of 650–1700 cm^{-1}.

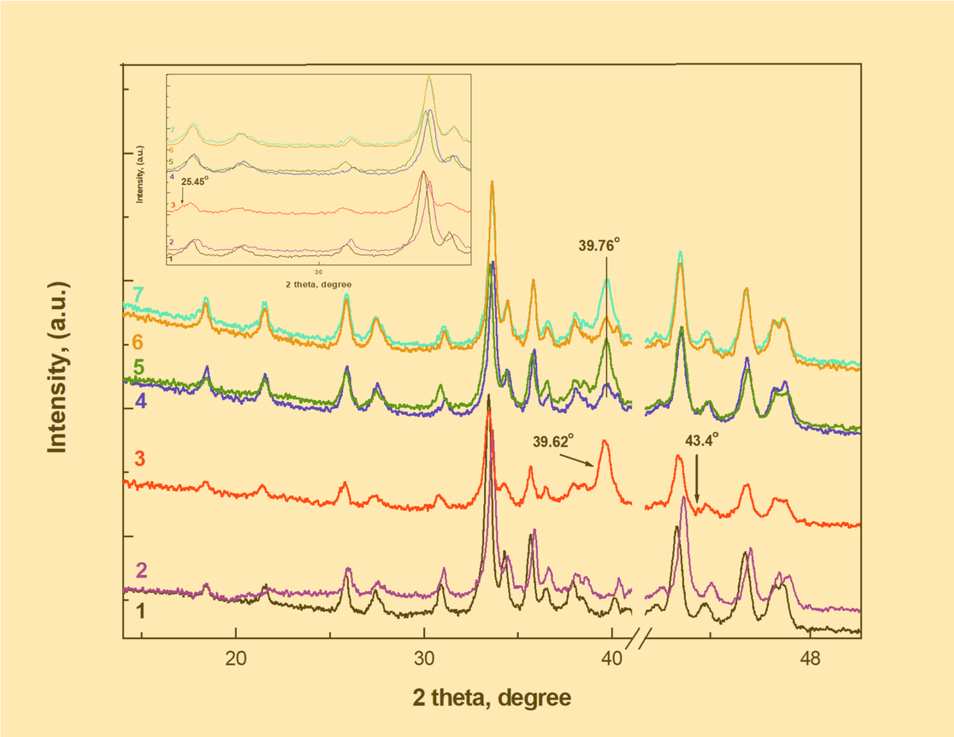

Figure 2. Diffraction patterns of PCPAB, MWCNTs, and PCPAB/MWCNT composites: PCPAB (1); PCPAB/ZnO (2); P/MWCNT-1 (3); P/MWCNT-2.1 (4); P/MWCNT-2.2 (5); P/MWCNT-3.1 (6); and P/MWCNT-3.2 (7).

Analysis of the IR spectra of PCPAB (spectrum 1, Figure 3a,b) and PCPAB/ZnO (spectrum 2, Figure 3a,b), converted to their neutral (main) form, also demonstrates the influence of ZnO on the formation of PCPAB. Calculating the ratio of the relative intensities of the bands of 1,4- and 1,2-substituted phenyl rings (D_{831}/D_{694}) in the spectra of the neutral form makes it possible to estimate indirectly the degree of polymerization of PCPAB and PCPAB/ZnO.

The data on absolute and relative intensities of these bands for a series of neutral PCPAB samples are presented in Table S1. The degree of polymerization in the neutral PCPAB polymer is higher than in PCPAB/ZnO. That is, the addition of Zn oxide led to an increase in the low molecular weight fraction in the polymer due to a decrease in the pH of the reaction solution [112], as well as to the appearance of additional reaction centers due to the higher dispersity of the reaction mixture. This resulted in a higher number of shorter polymer macromolecules and increased heterogeneity of the chemical structure [48,113].

A slight shift and splitting of the bands at 830 cm^{-1} and 712 cm^{-1} (spectrum 2, Figure 3b) due to deformation vibrations in the quinonoid rings of the PCPAB/ZnO sample indicates a change in the conformation of the polymer backbone. Therefore, it can be concluded that the addition of ZnO to the reaction system affects the polarization of the N-H, C-N, C=O, and aromatic C-C bonds and, consequently, the formation of PCPAB. The polarization of these bonds can occur due to the coordination of the Zn-O bond of the oxide to the O and H atoms of the monomer in the initial stage of the polymerization reaction, as shown in Scheme 3. This resulted in conformational diversity in the growing polymer chain, and the redistribution of the π-electron density in aromatic ring 1 also affected the degree of polymerization of the growing macromolecule.

https://doi.org/10.3390/polym18060000

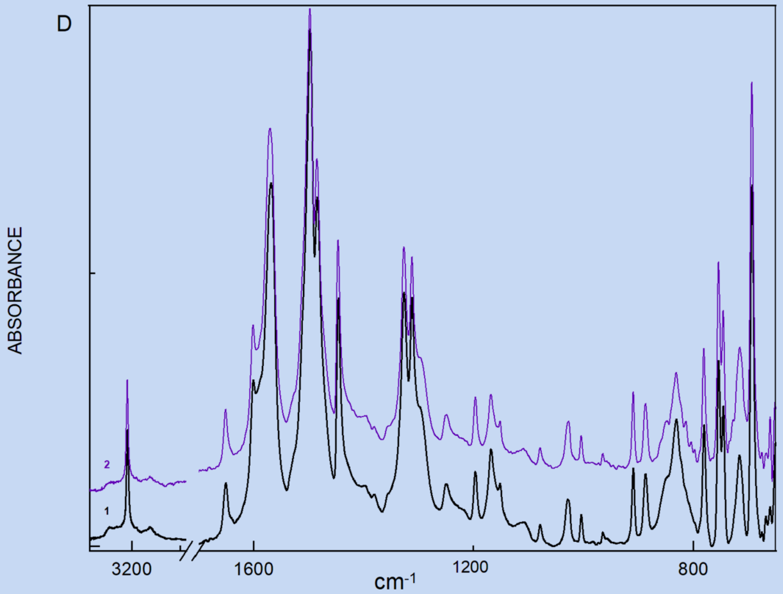

(**a**)

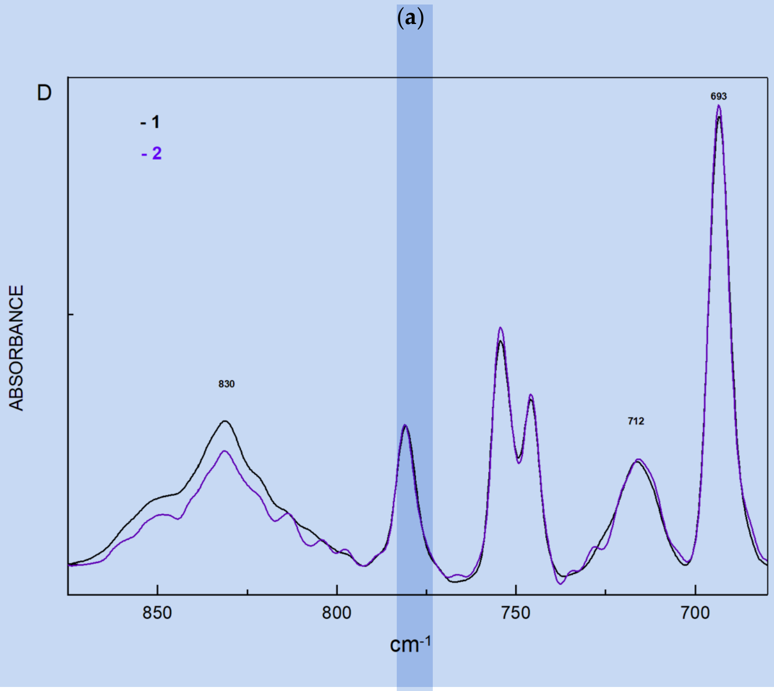

(**b**)

Figure 3. (**a**) IR spectra of samples (neutral form) obtained via oxidative polymerization of CPAB in HCl: PCPAB (1), in the presence of ZnO–PCPAB/ZnO (2). (**b**) Detailing in the region of 650–900 cm^{-1}.

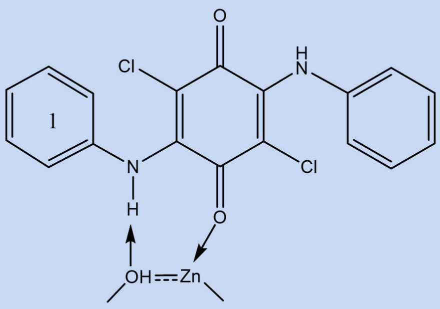

Scheme 3. Interaction of the monomer with metal oxide.

Figures 4 and 5 show the IR spectra of PCPAB, PCPAB/ZnO, and the composites based on them.

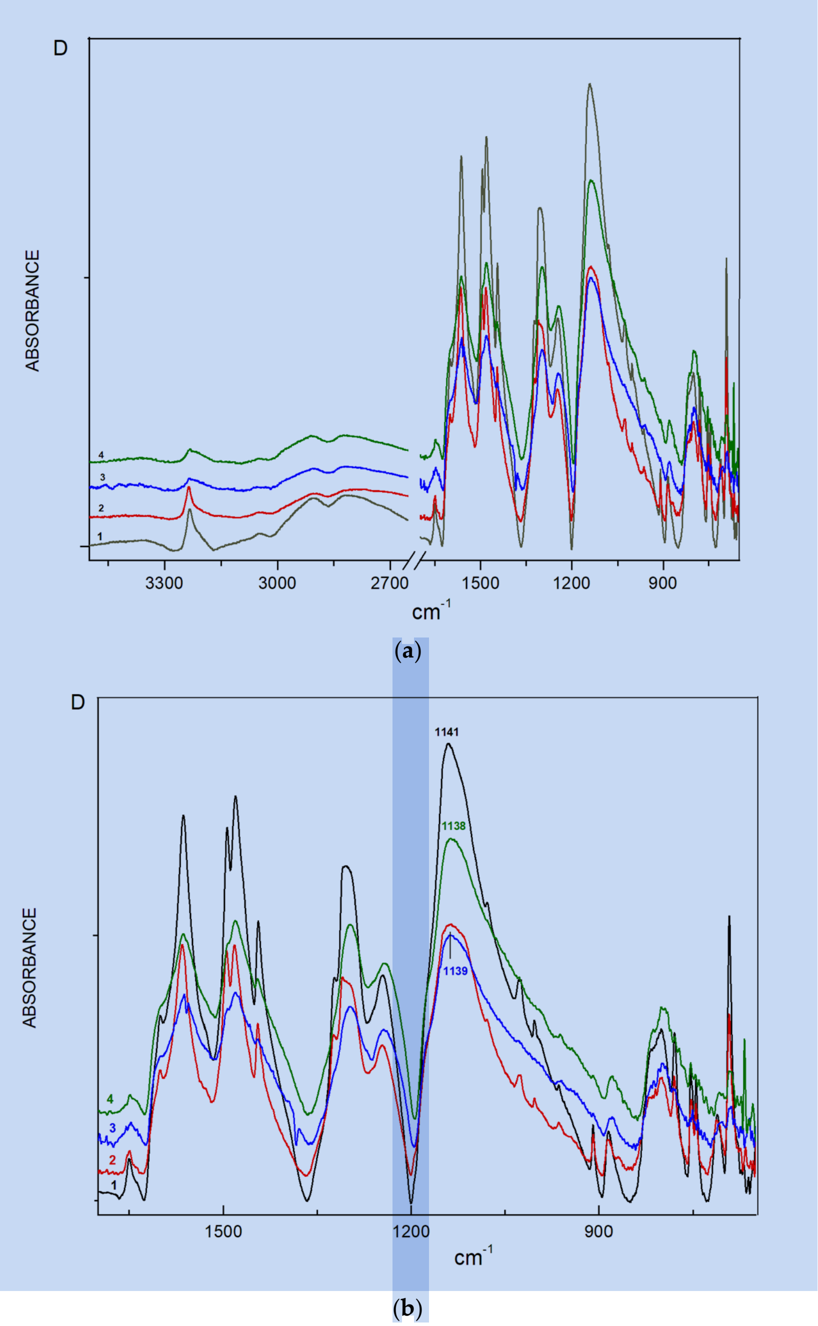

Figure 4. (**a**) IR spectra of samples (salt form) obtained via oxidative polymerization of CPAB in HCl: PCPAB (1), with MWCNTs P/MWCNT-1 (2); P/MWCNT-2.1 (3), P/MWCNT-2.2 (4). (**b**) Detailing in the region of 650–1700 cm^{-1}.

In the IR spectrum of P/MWCNT-1 (2 in Figure 4), all bands characteristic of PCPAB are observed, their intensities significantly reduced and the maxima shifted to lower frequencies. Additionally, a weak band associated with MWCNTs appears in the region of 867 cm^{-1} (Figure S1, spectrum 2). This is attributed to the interaction between the polymer and MWCNTs [15,114], which is confirmed by X-ray structural analysis data.

The diffraction pattern of P/MWCNT-1 (3, Figure 2) shows characteristic diffraction peaks of MWCNT 2 θ = 25.45° (shoulder), 39.62° (intense) and 43.4° (weak), corresponding to the (002), (111), and (101) planes, respectively [115]. All the reflection peaks of PCPAB are also observed, and the decrease in their height and slight shifts towards smaller angles

indicate an interaction of MWCNTs with the polymer and the formation of a hybrid composite material [116–118].

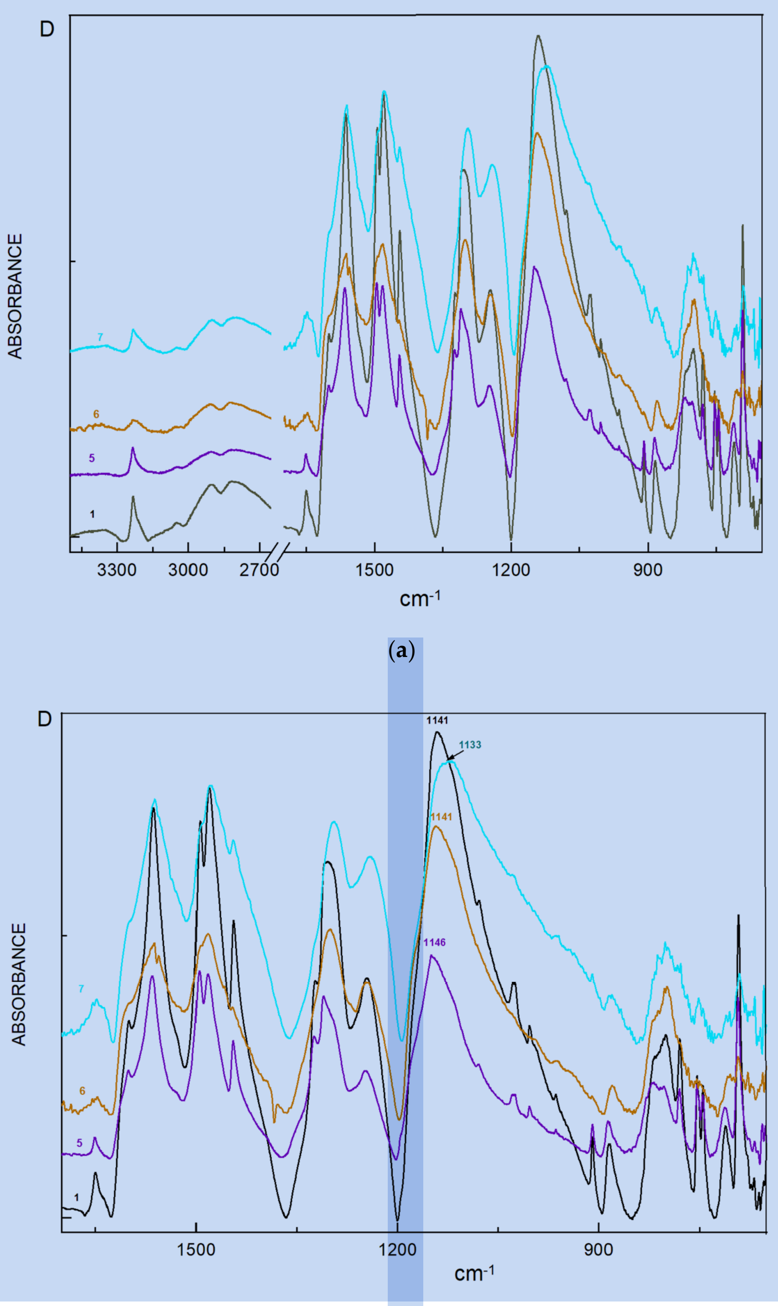

(b)

Figure 5. (**a**) IR spectra of samples (salt form) obtained via oxidative polymerization of CPAB in HCl: PCPAB (1), with the addition of ZnO PCPAB/ZnO (5), with MWCNTs and ZnO: P/MWCNT-3.1 (6), P/MWCNT-3.2 (7). (**b**) Detailing in the region of 650–1700 cm^{-1}.

In the IR spectra of the P/MWCNT-2.1, P/MWCNT-2.2 (spectra 3, 4; Figure 4), P/MWCNT-3.1, P/MWCNT-3.2 (spectra 6, 7; Figure 5) composite samples obtained via in situ oxidative polymerization, all bands of ammonium cations (in the region of ~801 and 1136–1139 cm^{-1}) appear more intense than in the spectrum of the initial PCPAB. This indicates an interaction of nanotubes with PCPAB through charge transfer between them, which, in turn, enhanced the non-covalent binding of ammonium cations to the polymer surface (Figure 6) [16,119].

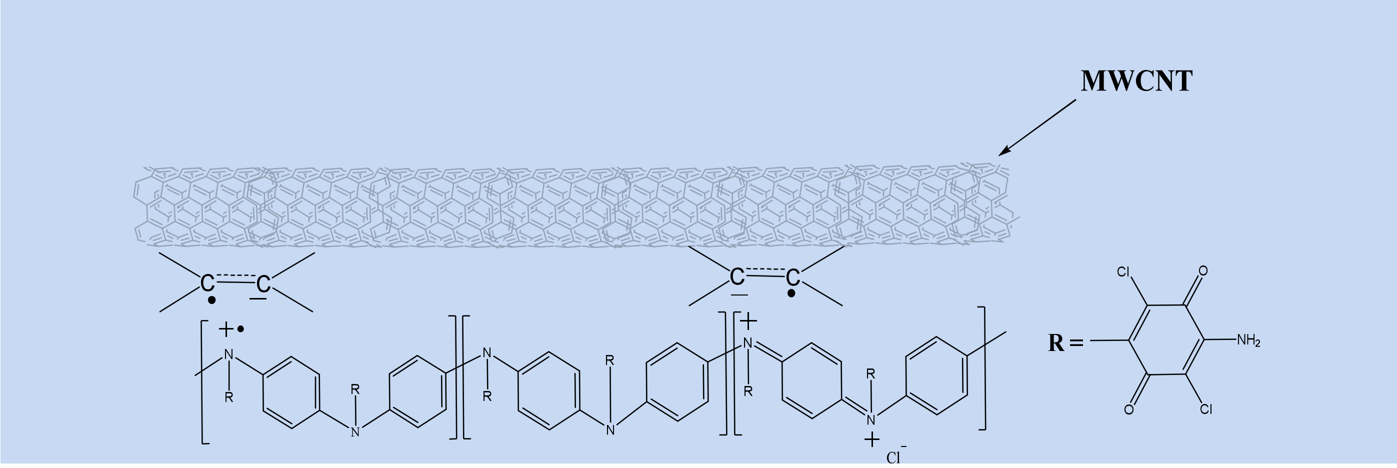

Figure 6. Proposed models of PCPAB–MWCNT interaction.

No bands from MWCNTs were observed, which indicates the incorporation of the filler into the polymer matrix [116]. The PCPAB bands in the spectra of these composites show significant changes: the bands at 1565 and 1482 cm^{-1} associated with the stretching vibrations of C=C bonds in N-substituted phenyl groups and at 1300 cm^{-1} associated with C-N bonds are shifted to longer wavelengths, as compared to PCPAB (spectrum 1, Figures 4 and 5). They become broader, and their intensity is further reduced, as compared to the spectra of PCPAB and P/MWCNT-1. The bands of the chloranil rings at 1650, 909 and 712 cm^{-1} (especially the first and the third, associated with stretching and deformation vibrations of the quinoid ring itself) were also split and slightly shifted towards longer wavelengths.

Figure 7 presents the spectra of PCPAB, P/MWCNT-2.2, and P/MWCNT-3.2 in the range of 675–870 cm^{-1}, as it is the deformation of the C-C-H angles in the aromatic rings that is most sensitive to the conformation of the chain containing aromatic rings. A new broad, split, low-intensity band appears in the region of 750 cm^{-1} (Figure 7, spectra 2, 3) instead of the doublet at 745 and 754 cm^{-1} in the PCPAB spectrum (Figure 7, spectrum 1) associated with δCCH deformation vibrations in di-substituted aromatic rings. The bands associated with out-of-plane δC-C-H deformation vibrations of aromatic rings—at 693 cm^{-1} for monosubstituted and at 820 cm^{-1} for 1,4-substituted rings, overlapping with the band at 800 cm^{-1} from ammonium cations—also showed significant splitting and shifted towards longer wavelengths. The decrease in band intensity is caused by the adhesion, first of ions of the monomer and MWCNTs due to the formation of a weak charge-transfer complex between them (Figure 8), and then of growing polymer chains on the MWCNT surface, which restricts their movement along it and, consequently, limits possible vibrational modes within the polymer (Figure S2) [116,120]. As a result, the spectra of composites obtained by Methods 2 and 3 demonstrated significant conformational alterations of the polymer macromolecules under the influence of the MWCNT surface, which indicates the interaction between them.

In the spectrum of P/MWCNT-3.2 (Figure 7, spectrum 3), an even greater shift to the long-wavelength region is observed, which indicates an increase in charge delocalization between MWCNTs and PCPAB due to a stronger interaction between the filler surface and the polymer [115,121]. This is due to the fact that ZnO powder increases the number of nucleation points of polymer chains in the immediate vicinity of carbon nanotubes in the first stage of polymerization. Then, with the addition of acid and oxidizing agent, the growth of macromolecules begins with the gradual formation of Zn^{2+} ions during the dissolution of zinc oxide in an acidic medium. As a result, due to the formation of shorter PCPAB macromolecules in the presence of MWCNTs [48,122] and the interchain complexation between the polymer chains and zinc ions (Scheme 4), a denser and more uniform polymer layer is formed around the nanotubes [34–36]. Thus, ZnO and its salt,

during the composite preparation process, enhance the role of MWCNT as a template and encourage more compact polymer chain packing. This contributes to an increase in the crystallinity of PCPAB, as confirmed by X-ray diffraction analysis data.

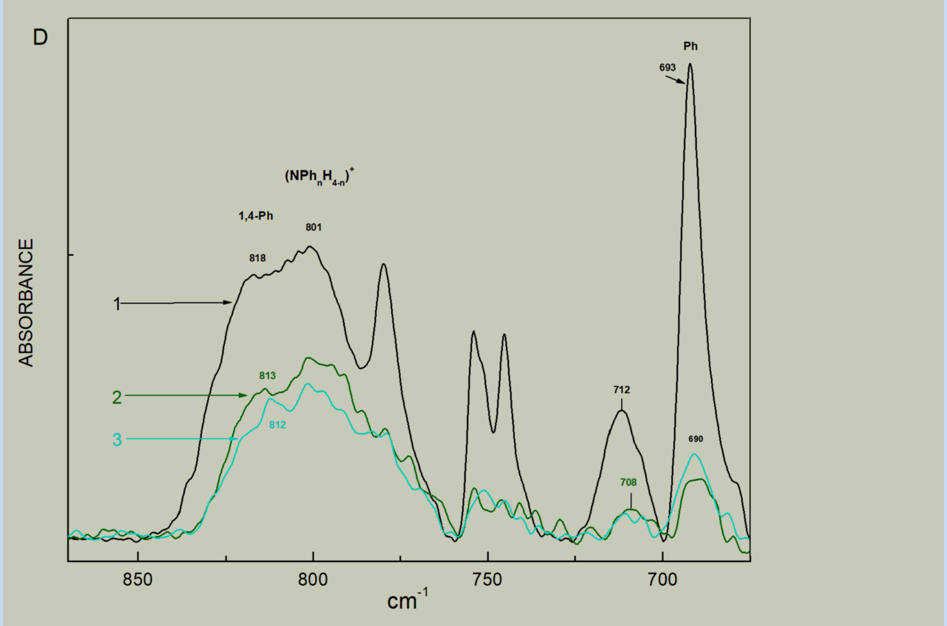

Figure 7. IR spectra of PCPAB (1), P/MWCNT-2.2 (2) and P/MWCNT-3.2 (3) composites.

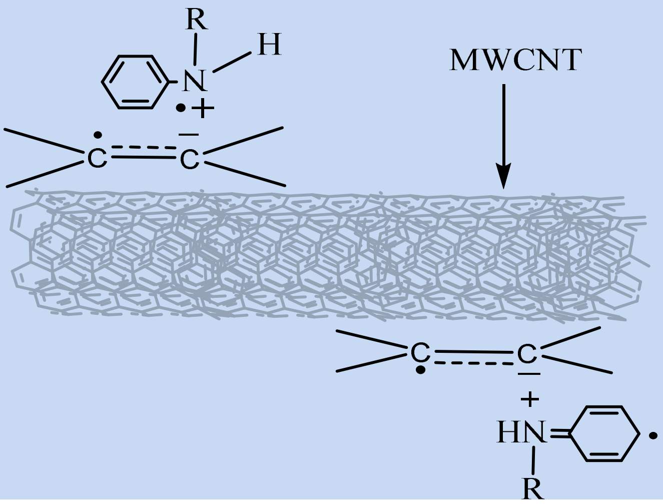

Figure 8. Proposed models of CPAB–MWCNT interaction.

The X-ray diffraction patterns of P/MWCNT-2.1, P/MWCNT-2.2, P/MWCNT-3.1 and P/MWCNT-3.2 composites (4–7; Figure 2) demonstrate characteristic peaks of PCPAB without any additional reflection peaks, indicating a non-covalent type of bond between the polymer and MWCNTs in the composite. No peaks of MWCNTs were observed in these samples in the region of 2θ = 25.5° and 43.5°, which indicates the incorporation of the filler into the polymer matrix. A decrease in the intensity of individual PCPAB bands and a slight shift of 2θ peaks—of both the polymer and MWCNTs—to the region of 2θ = 39.76° may be attributed to the doping effect of the carbon filler on PCPAB, as illustrated in Figure 6. This leads to an increase in charge delocalization along the polymer chains [115,121,123]. At the same time, some peaks of PCPAB—which are low-intensity and broad in the initial polymer due to scattering from polymer chains at the interplanar distance (diffraction pattern 1, Figure 2)—become narrower and more intense in composites

(diffraction patterns 4–7, Figure 2). This indicates an increase in the degree of polymer crystallinity due to the formation of a denser and more regular packing of polymer chains along the MWCNTs [124]. All of this confirms the formation of a polymer coating on the surface of the carbon filler during in situ oxidative polymerization in the presence of MWCNTs and the π-π* interaction between PCPAB and carbon aromatic rings on the surface of MWCNTs [114,120,125].

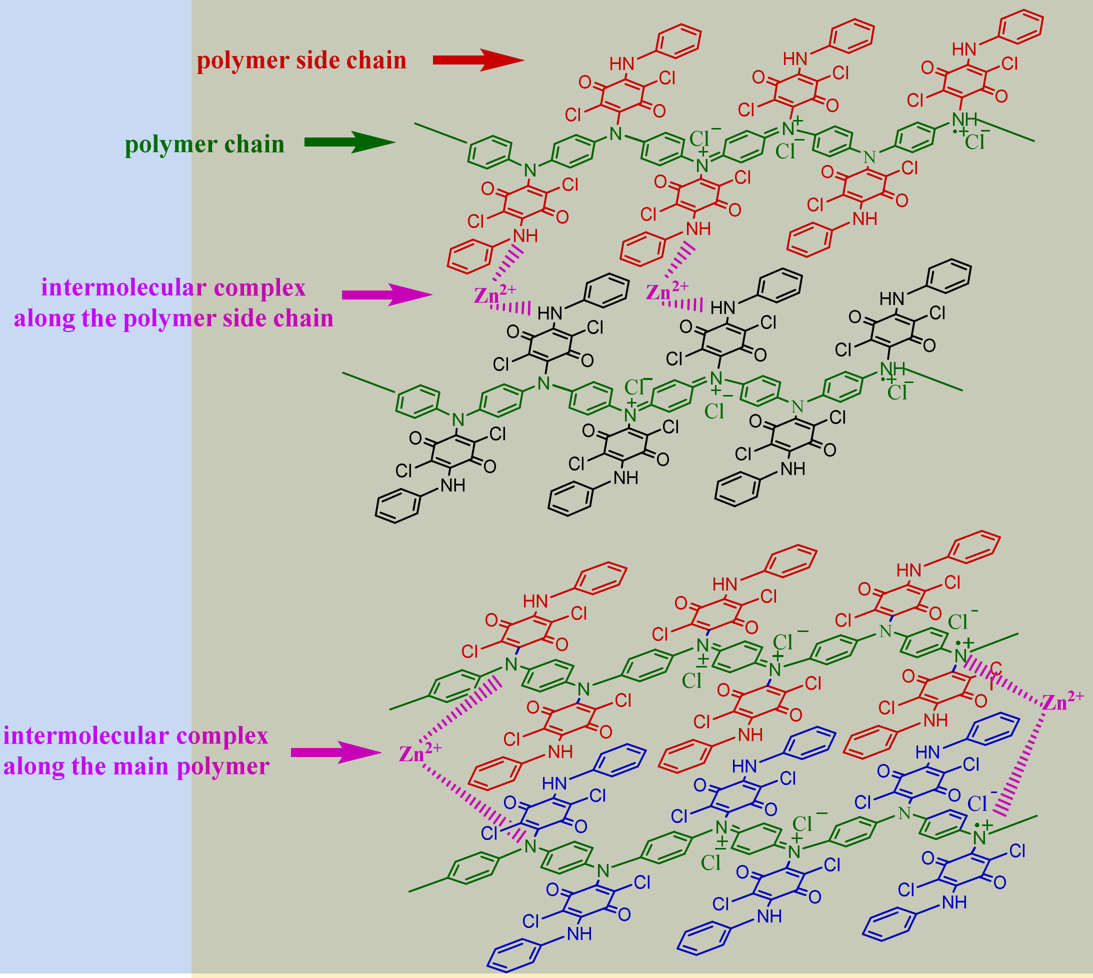

Scheme 4. Schematic representation of possible variants of intermolecular complexes of PCPAB with Zn^{2+}.

In P/MWCNT-2.2 (diffraction pattern 5, Figure 2), an increase in the intensity of the MWCNTs reflection peak (2θ = 39.76°) and a slight decrease in intensity and a shift to the region of low 2θ values of individual peaks of PDAHB were observed compared to P/MWCNT-2.1 (diffraction pattern 4, Figure 2). This is attributed to an increase in the MWCNT content in the polymer matrix and the associated disruption of the regular packing of polymer chains [58,126].

In the samples P/MWCNT-3.1 and P/MWCNT-3.2 (diffraction patterns 6 and 7, Figure 2), almost complete coincidence in the intensity of all PCPAB reflection peaks was observed. This indicates that the use of ZnO mitigates the increase in disorder in the packing of polymer macromolecules relative to one another with the increase in MWCNT content in the composite, as was observed in P/MWCNT-2.1 and P/MWCNT-2.2 samples. In the reaction mixture preparation stage, ZnO powder, localizing along MWCNTs, prevents their aggregation and ensures a more uniform distribution of nanotubes in the reaction mixture and an increase in their specific surface area. As a result, the monomer concentration

near MWCNTs grows, as zinc oxide particles can act as additional reaction centers for the nucleation of polymer chains in close proximity to nanotubes. This, in turn, facilitates the formation of a more compact and regular packing of a larger number of PCPAB chains around MWCNTs, and increases the thickness and density of the polymer layer near and on the surface of the carbon filler, preventing its agglomeration in the composite. It should be noted that peaks of Zn compounds were not observed on the diffractograms of the samples PCPAB/ZnO, P/MWCNT-3.1 and P/MWCNT-3.2 [84,127], since they are highly soluble in the acidic medium of the reaction solution and are almost completely removed with it during filtration. This is confirmed by atomic emission spectroscopy data (Table 1). However, these compounds have an impact on the formation of the composite material structure.

The influence of MWCNTs and ZnO on the formation of PCPAB during in situ oxidative polymerization of CPAB is also evident from the analysis of the kinetic curves of the change in redox potential, presented in Figure 9.

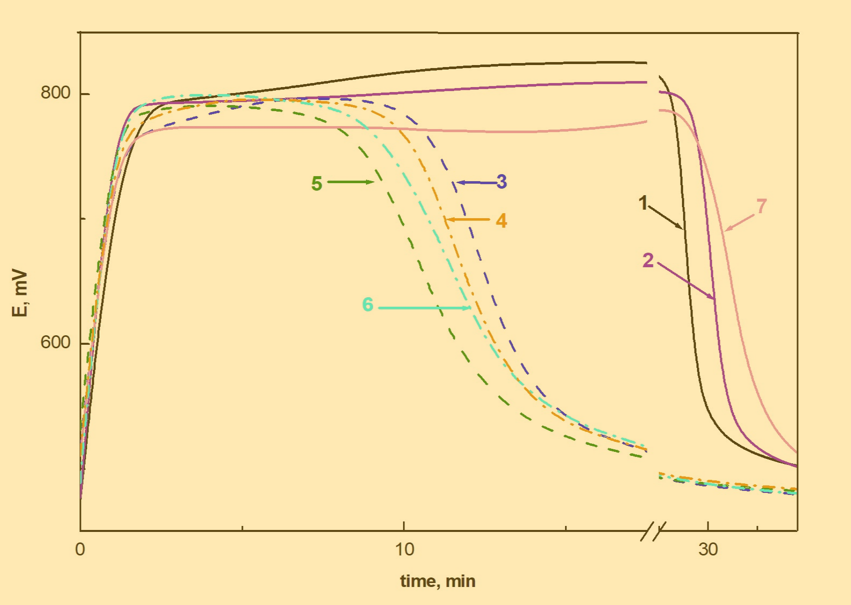

Figure 9. Changes in redox potential over time during oxidative polymerization of CPAB: without additives PCPAB (1); +25% ZnO PCPAB/ZnO (2); +45% ZnO (7); P/MWCNT-2.1 (dashed line 3); MWCNT P/MWCNT-3.1 (dot-dash line 4); P/MWCNT-2.2 (dashed line 5); P/MWCNT-3.2 (dot-dash line 6).

When ZnO is added to the reaction mixture in the absence of MWCNTs, a slight increase in the induction period of oxidative polymerization of CPAB and a decrease in the reaction rate (curves 1, 2, 7) are observed. This is proportional to the amount of oxide due to a decrease in the pH of the reaction solution [100]. In the presence of ZnO and/or MWCNTs, the kinetics of the process depends mainly on the dispersity of the reaction mixture. The 2–2.5 times decrease in the induction period in composite samples (curves 3–6) is attributed to an increase in the number of polymerization reaction centers [48,128]. Thus, the addition of ZnO and/or MWCNT affects the formation process of PCPAB and composites based on it.

Figure 10 shows the Raman spectra of PCPAB samples and its composites with MWCNTs in salt form.

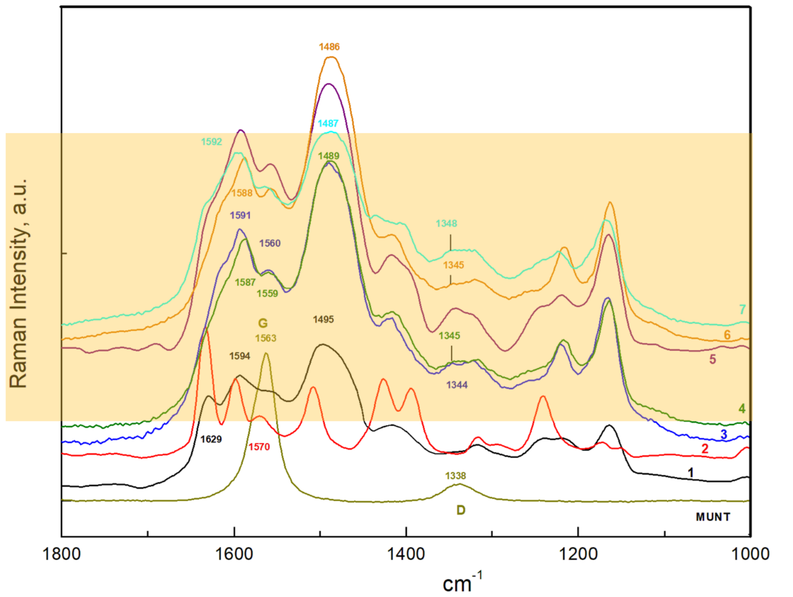

Figure 10. Raman spectra of samples obtained via oxidative polymerization of CPAB in HCl:MWCNTs; PCPAB (1); P/MWCNT-1 (2); P/MWCNT-2.1 (3); P/MWCNT-2.2 (4); PCPAB/ZnO (5); P/MWCNT-3.1 (6); P/MWCNT-3.2 (7).

In the Raman spectrum of PCPAB in its salt form (Figure 10, spectrum 1), two strong signals at 1593 and 1495 cm^{-1} correspond to the asymmetric and symmetric vibrations of the C=C bonds in the aromatic rings of the polymer. Each of these signals had a well-defined long-wavelength shoulder: 1558 cm^{-1} for the first one and 1415 cm^{-1} for the second one. This indicates the presence of two types of aromatic rings in the samples: rings with vibrational modes at 1593 and 1492 cm^{-1} have a higher π-electron density, while rings characterized by signals at 1557 and 1418 cm^{-1} have a lower π-electron density due to delocalization via an electron pair of nitrogen atoms with the HCl dopant present in the salt form of the polymer. This non-covalent bonding resulted in the appearance of chain segments with an uneven distribution of electron density in the conjugation system and, consequently, to the emergence of new signals from C=C bonds in Raman spectra [100,129–131]. Bands in the region of 1165 cm^{-1} are related to the deformation in-plane vibrations of the C-H bond in quinoid rings, bands at 1240/1265 cm^{-1} and 1310 cm^{-1} are associated with stretching vibrations of the C-N bond in benzene rings and the $C \approx N^{\bullet+}$ bond in the bipolaron structure of the polymer chain ($>N^{+}=Qu=N^{+}<$) [132–135].

The spectrum of the PCPAB/ZnO sample (Figure 10, spectrum 5) showed an increased intensity of ν_{ass} bands (1600–1594 cm^{-1}) along with a stronger splitting of all bands, which is attributed to a decrease in the doping degree and heterogeneity of the chemical structure [136]. According to the selection rules for Raman spectra, the position of a peak and especially its intensity also depends on the bond polarizability. This indicates that zinc oxide significantly changes the polarizability of C–C bonds in the aromatic rings of CPAB in the first stage of polymerization, thereby affecting significantly structural and especially electronic characteristics of aromatic rings in the growing polymer chain (Scheme 3) [129,130].

The spectrum of P/MWCNT-1 (Figure 10, spectrum 2) is not an additive superposition of the Raman signals from PCPAB and MWCNTs, which indicates the formation of a composite. All signals from the polymer's aromatic rings have changed relative intensities dramatically compared to the signals in the Raman spectrum of PCPAB (Figure 10, spectrum 1) and have shifted towards higher frequencies. This can be attributed to a combination of factors: a decrease in the degree of PCPAB doping and interactions between the components

https://doi.org/10.3390/polym18060000

during the formation of the composite material. Medium intensity bands at 1425 and 1394 cm^{-1} are prominent in the spectrum of P/MWCNT-1. They may result from the formation of cross-linked PCPAB fragments. Since ultrasonic treatment of an aqueous suspension of PCPAB and MWCNTs during the preparation of PCPAB/MWCNT-1 involves cavitation and local overheating processes [137], this may induce both a decrease in the degree of PCPAB doping, and the rupture of individual bonds with the formation of low-molecular "fragments" and "phenazine-like" structures cross-linked due to interchain interaction [102,138,139]. The G signal, which characterizes the sp^2 carbon atoms of the filler, shifted in the spectrum of P/MWCNT-1 to the short-wave region by 7 cm^{-1} (to 1570 cm^{-1}) compared to the spectrum of MWCNTs alone. This behavior of the signals from vibrational modes of π-electrons in the aromatic rings of PCPAB and MWCNTs is caused by a decrease in the particle size in the sample prepared under ultrasonic treatment [58], resulting in a decrease in the degree of π-conjugation of electrons in aromatic structures, accompanied by a frequency shift to the short-wave region [102].

Peak D in the spectrum of P/MWCNT-1 appeared in the longer-wavelength region of 1317 cm^{-1}—compared to 1338 cm^{-1} in the Raman spectrum of MWCNT. Signal D, associated with the vibrational modes of the terminal sp^3 carbon atoms, reflects the presence of defects at the edges of the particles [120,140]. An increase in the number of such defects should be accompanied by an increase in the signal intensity and its shift to the longer wavelengths, which is indeed observed in the spectrum of P/MWCNT-1 due to the impact of ultrasonic treatment throughout the entire process of composite preparation.

In the spectra of the P/MWCNT-2.1 and P/MWCNT-2.2 composites (Figure 10, spectra 3 and 4), obtained in situ in the presence of MWCNTs, all frequencies of the vibrational modes of the C=C bonds in the aromatic rings of PCPAB (1629, 1594, 1498, 1423 cm^{-1}) (Figure 10, spectrum 1), in contrast to the spectrum of P/MWCNT-1, have shifted to the low frequency range. The greatest shift was observed for the frequency of 1498 cm^{-1} associated with symmetric stretching vibrations of the C=C bonds, which are most sensitive to bond polarization. The Raman spectra of these composites are virtually indistinguishable from the spectrum of PCPAB (Figure 10, spectrum 1). The presence of MWCNTs in them is indicated only by a weak signal D in MWCNTs, shifted to the short-wavelength region by 7 cm^{-1} (~1345 cm^{-1}). The G signal coincides with the broad vibrational mode ν_{ass} (C=C(Ph + Qu)) in the polymer spectrum in the range of 1565–1593 cm^{-1}. Its shift to lower frequencies also indicates the interaction of the polymer component with the carbon filler. Thus, the IR and Raman spectroscopy data both indicate the formation of a PCPAB layer on the surface during the growth of polymer chains around carbon tubes. This coating prevents the aggregation of MWCNTs and ensures a uniform distribution of the tubes in the polymer matrix [120,123].

The Raman spectra of P/MWCNT-3.1 and P/MWCNT-3.2 in Figure 10 (spectra 6, 7) are similar to the spectra of P/MWCNT-2.1 and P/MWCNT-2.2 (spectra 3, 4) in the nature of frequency shifts from vibrational modes of the C=C bonds in aromatic rings. However, in P/MWCNT-2.1 and P/MWCNT-2.2 frequency intensities are almost independent of the MWCNT content, whereas in P/MWCNT-3.1 and P/MWCNT-3.2 frequency intensities from ν_{ass} (1600 cm^{-1}) and ν_s (1498 cm^{-1}) change. This indicates a higher efficiency of charge transfer processes between PCPAB and MWCNTs, which in this case depends on the filler content in the composite and is associated with a stronger interaction between nanotubes, ZnO and growing polymer chains in the initial stage of synthesis [81,141]. This is also ensured by the formation of a denser polymer coating on the surface of MWCNTs due to complexation between polymer chains and zinc ions (Scheme 4) [36,44], which is confirmed by electron microscopy data.

The conditions for the formation of nanocomposites based on PCPAB and MWCNTs determine not only their composition and structure but also their morphology. Figure 11 shows SEM micrographs of PCPAB, PCPAB/ZnO, and its hybrid nanocomposites. PCPAB (Figure 11a) is characterized by a relief morphology formed by large aggregates of irregularly shaped PCPAB particles. In PCPAB/ZnO (Figure 11b), alongside agglomerated particles of indefinite shape, clusters of fibril-like structures are observed, which is associated with the heterogeneity of the chemical structure and confirms the IR spectroscopy data.

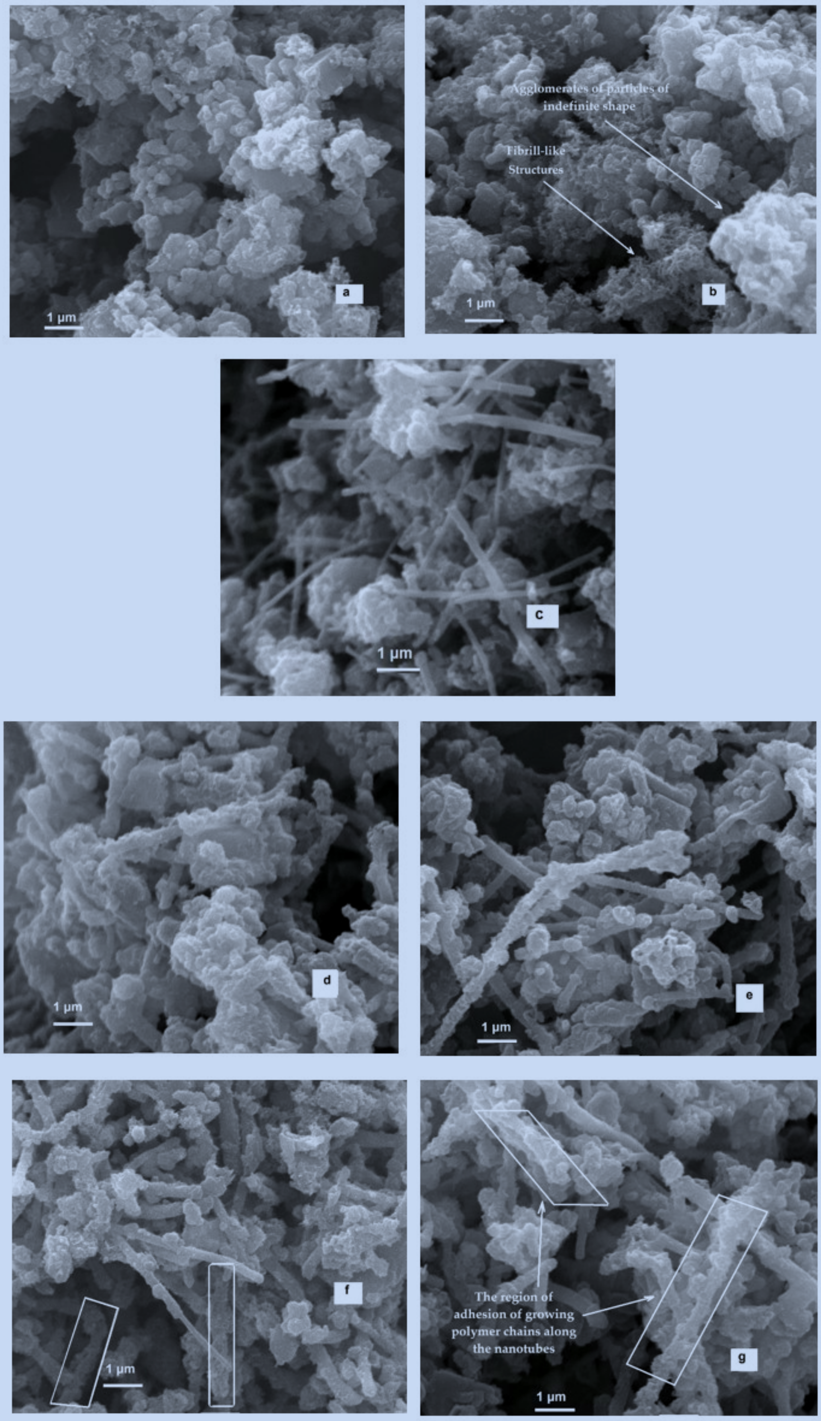

Figure 11. Electron microscopic image of PCPAB, PCPAB/ZnO and its composites with MWCNTs: PCPAB (**a**); PCPAB/ZnO (**b**); P/MWCNT-1 (**c**); P/MWCNT-2.1 (**d**); P/MWCNT-2.2 (**e**); P/MWCNT-3.1 (**f**); P/MWCNT-3.2 (**g**).

https://doi.org/10.3390/polym18060000

Figure 11c demonstrates the presence of nanotubes in the P/MWCNT-1 nanocomposite. Through ultrasonic treatment, these nanotubes are distributed between the PCPAB agglomerates. When obtaining nanocomposites via Method 2, a polymer layer of fine texture was formed on the surface of the template, as demonstrated by Figure 11d–g. In P/MWCNT-2.1 (Figure 11d), a high content of PCPAB formed in the bulk of the reaction solution is observed, and nanotubes are immersed in its agglomerates. The amount of bulk PCPAB decreases noticeably with an increase in the filler weight in P/MWCNT-2.2 (Figure 11d,e). In P/MWCNT-3.1 and P/MWCNT-3.2 (Figure 11f,g), respectively, the thickness of tubes coated with the polymer layer increases. When the MWCNT content in P/MWCNT-3.2 increases, a decrease in the amount of bulk PCPAB is also observed, as compared to P/MWCNT-3.1. However, unlike in the case of P/MWCNT-2.1 and P/MWCNT-2.2, its localization (sticking) is detected mainly along the entire length of nanotubes, as shown in Figure 11f,g. This also aligns with our thesis that ZnO powder, during the initial stages of composite preparation, adsorbs onto the surface of MWCNTs, thereby increasing the concentration of nucleation sites for polymer chains near the nanotube surface and, as a consequence, the thickness of the polymer coating. In general, samples obtained by Method 3 represent a material with a denser packing of nanotubes coated with a polymer layer relative to each other.

Based on IR, Raman, XRD, and SEM data, it can be concluded that all samples based on PCPAB and MWCNTs, obtained regardless of the preparation method, are hybrid composite materials exhibiting a fairly strong interaction between the components. The strength of this interaction decreases in the following order: P/MWCNT-3 > P/MWCNT-2 > P/MWCNT-1. In P/MWCNT-1, this is associated with the non-uniform distribution of nanotubes within the sample. As a result of Methods 2 and 3, nanocomposites are obtained in which the polymer coating is not covalently bonded to the surface of the MWCNTs. The use of ZnO promotes the formation of a denser coating on the MWCNT surface due to the localization of the growing polymer chain in close proximity to the nanotubes and interchain complexation between the growing chains of PCPAB macromolecules with the participation of Zn^{2+} ions formed during the polymerization of PCPAB in an aqueous HCl solution in the presence of ZnO. At the same time, the interaction of CPAB with MWCNTs and, consequently, the charge delocalization between them improves.

3.2. *Electrical Conductivity of Composites Based on PCPAB and MWCNTs*

The dependence of electrical conductivity σ_{ac} on frequency is described by the following equation [142,143]:

$$\sigma_{ac} = \sigma_{dc} + A\omega^n,$$

where $\omega = 2\pi f$ is angular frequency, σ_{dc} is the frequency-independent part of conductivity, n—is the exponential factor ($0 \leq n \geq 1$), and A is a temperature-dependent coefficient.

Figure 12 shows the dependence of the electrical conductivity of PCPAB and PCPAB-based composites on frequency in the frequency range of 25–10^6 Hz.

Table 2 provides the values of σ_{ac}, σ_{dc}, and n calculated by approximating experimental data. The nature of the frequency dependence of the electrical conductivity of PCPAB and PCPAB/ZnO is typical for conducting polymers [143,144]. The values of n indicate a hopping conductivity mechanism (Table 2). The lower electrical conductivity in PCPAB/ZnO is associated with a decrease in the degree of polymerization and the degree of doping of the polymer, as was shown in Section 3.1.

Based on the values of parameter n for PCPAB/MWCNT composites, the conductivity mechanism, regardless of their formation method, is predominantly hopping [142–144]. In composites based on PCPAB and MWCNTs, the electrical conductivity values increase by 6–12 times compared to the electrical conductivity of PCPAB, depending on the filler

content. This is determined by the degree of percolation introduced by MWCNTs. Due to their high conductivity and high aspect ratio, nanotubes can act as bridges between conductive polymer particles, leading to an increase in the electrical conductivity of the composite [126,145]. The lower conductivity value of the P/MWCNT-1 composite obtained by mixing the prepared polymer and MWCNTs, compared to the other composites, is due to an increase in the number of defective structures, a decrease in the degree of π-conjugation of PCPAB and the non-uniform distribution of the filler in the PCPAB matrix (Figure 11c) as a result of ultrasonic treatment.

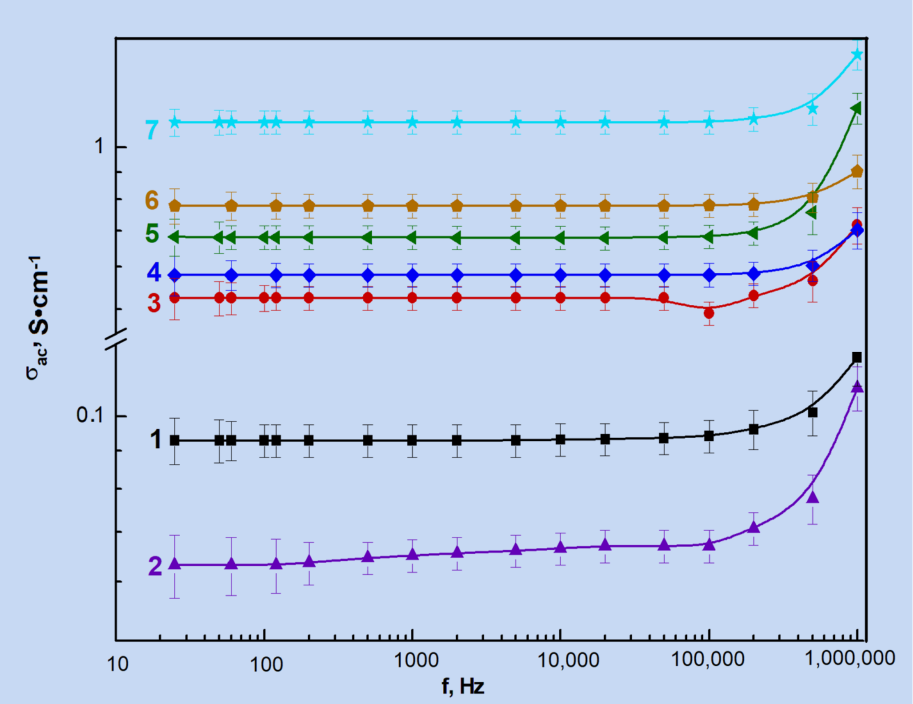

Figure 12. Current frequency dependence of conductivity: PCPAB (1); PCPAB/ZnO (2); P/MWCNT-1 (3); P/MWCNT-2.1 (4); P/MWCNT-2.2 (5); P/MWCNT-3.1 (6); P/MWCNT-3.2 (7).

Table 2. Electrical conductivity of PCPAB/MWCNT nanocomposites.

SAMPLE	σ_{dc}, $S{\cdot}cm^{-1}$	σ_{ac} (25 Hz), $S{\cdot}cm^{-1}$	σ_{ac} (1 MHz), $S{\cdot}cm^{-1}$	n
PCPAB	9.2×10^{-2}	9.3×10^{-2}	1.2×10^{-1}	0.99
PCPAB/ZnO	6.1×10^{-2}	6.2×10^{-2}	1.0×10^{-1}	0.91
P/MWCNT-1	5.2×10^{-1}	5.3×10^{-1}	7×10^{-1}	0.95
P/MWCNT-2.1	5.7×10^{-1}	5.8×10^{-1}	7×10^{-1}	0.84
P/MWCNT-2.2	6.7×10^{-1}	6.8×10^{-1}	1.18	0.88
P/MWCNT-3.1	7.8×10^{-1}	7.8×10^{-1}	9×10^{-1}	0.89
P/MWCNT-3.2	1.12	1.13	1.52	0.9

The electrical conductivity of P/MWCNT-3.1 and P/MWCNT-3.2 is higher than that of P/MWCNT-2.1 and P/MWCNT-2.2 (Figure 13), which confirms the formation of a denser and more uniform polymer coating on the MWCNT surface and a more homogeneous distribution of the filler within the composite. This enhances the interconnectivity between PCPAB chains and boosts the efficiency of charge transfer processes between composite components. Thus, depending on the method of preparation and content of MWCNTs, the electrical conductivity of the nanocomposites increased by 7.3 times compared to PCPAB,

https://doi.org/10.3390/polym18060000

reaching 0.67 S·cm^{-1}. The addition of ZnO during their preparation further enhances the electrical conductivity of the samples by an additional 60%, to 1.1 S·cm^{-1}.

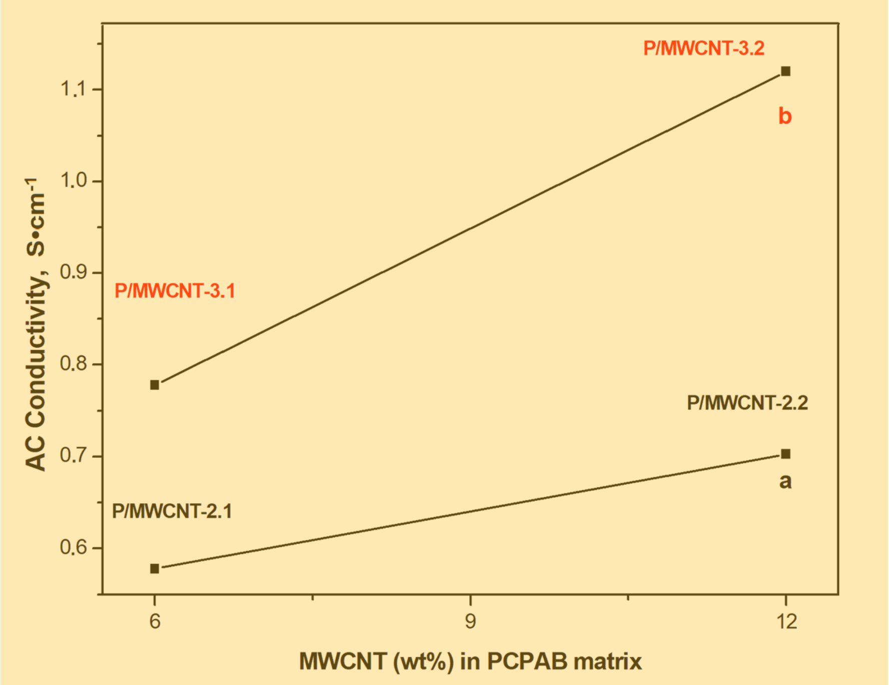

Figure 13. Electrical conductivity dependence of composites obtained via method 2 (a) and method 3 (b) on the content of MWCNTs.

Based on the PCPAB/MWCNTs nanocomposites, electrodes for supercapacitors were obtained, which are a glassy carbon substrate with a layer of electroactive nanocomposite applied to it. The electrochemical behavior of PCPAB/MWCNT nanocomposites in protic (1 M H_2SO_4) and aprotic (1 M $LiClO_4$ in propylene carbonate) electrolytes was studied depending on the MWCNTs content [146]. An increase in the specific electrochemical specific capacity of $C_{sp.}$ nanocomposites was shown in comparison with PCPAB due to an increase in the conductivity of the electrode material with a larger surface area. Both components of the nanocomposite contribute to the electrochemical capacitance by charging the electrical double layer of the MWCNTs (double-layer capacitance) and by rapidly repetitive redox reactions of the polymer (Faraday pseudocapacitance) [147]. An extreme dependence of electrochemical capacity on the carbon nanoparticle content was demonstrated. Maximum $C_{sp.}$ values were achieved with a MWCNTs content in the nanocomposite of 14% by weight in an aqueous acidic electrolyte (140 F·g^{-1}, I = 0.4 A·g^{-1}) and 17% by weight in an organic electrolyte (46 F·g^{-1}, I = 0.2–1.0 A·g^{-1}) at a potential scan rate of 0.05 V·s^{-1}). The Coulomb efficiency η was 99–100%. The increase in capacity was mainly due to the increase in conductivity due to the formation of a conductive framework of MWCNTs coated with a polymer shell. The decrease in the value of $C_{sp.}$ with an increase in the concentration of MWCNTs was probably associated with a decrease in the proportion of electroactive polymer and a change in the morphology of the nanocomposite.

The available literature data on the electrochemical characteristics of hybrid PCS/MWCNTs nanocomposites primarily describe PANI-based nanocomposites. Analysis of the electrochemical properties revealed that the quantitative values of electrochemical capacity are highly dependent on the method of depositing the electroactive layer on the substrate (electrochemically or from a solution (suspension)), the type of electrochemical cell, the nature of the electrolyte, the cycling mode, the potential sweep rate, the MWCNTs content and their functionalization, etc. The values of the surface capacitance of the PCS/MWCNT based electrodes varied from 12 to 530 F·g^{-1} [148–154]. The surface capacitance of the PANI/MWCNT nanocomposite measured

in [148] was only 0.6 F·cm^{-2} at a current density of 0.2 A·cm^{-2}. The above results from the study of PCPAB/MWCNT nanocomposites are consistent with the available literature data. The low values of $C_{sp.}$ are explained by the fact that in the first stage, we used a smooth glassy carbon substrate with a very low specific surface area. A significant advantage of PCPAB/MWCNTs nanocomposites is the short time (less than 24 h) it takes to reach steady-state, compared to 5 days for PANI/MWCNTs nanocomposites [149]. This is due to the high packing density of the PANI polymer chains, which is due to their interaction not only with the MWCNTs but also with each other. As a result, counterion transport within the bulk of the electrode material was hampered by electrolyte deficiency. The bulky chloranil substituents in the PCAPB/MWCNTs nanocomposites increased the free volume and improved electrolyte diffusion. The work is not complete, and as we continue to develop it, we will test substrates with highly developed surfaces and also vary the carbon component. We have already achieved excellent results with nanocomposites based on PCAPB and graphene oxide, deposited on an anodized graphite foil substrate, in a proton electrolyte. The specific electrochemical capacity was 842 F·g^{-1}. This value decreased by only 4% with an increase in discharge current from 0.1 to 1.1 A·g^{-1} [101].

3.3. Thermal Stability of Nanocomposites Based on PCPAB and MWCNTs

Figure 14 and Figure S3 show the temperature dependences of mass change (TGA) and differential thermogravimetric (DTG) curves for the PCPAB polymer and PCPAB/MWCNT nanocomposites obtained by various methods. Thermogravimetric analysis results are presented in Table 3.

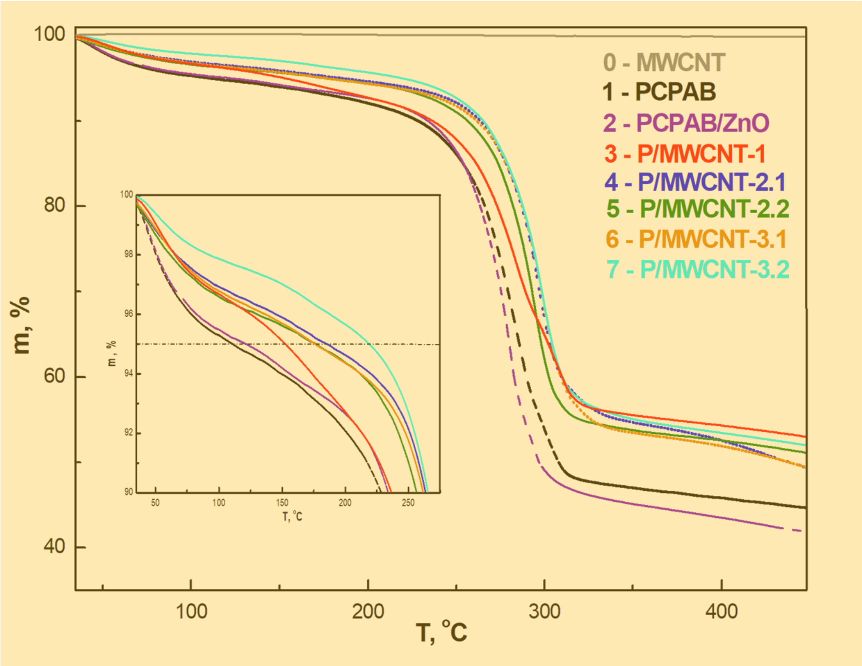

Figure 14. DTA curves—MWCNT (0); PCPAB (1); PCPAB/ZnO (2); P/MWCNT-1 (3); P/MWCNT-2.1 (4); P/MWCNT-2.2 (5); P/MWCNT-3.1 (6); P/MWCNT-3.2 (7).

Figure 14 shows the thermograms of PCPAB, PCPAB/ZnO, and composites based on PCPAB and MWCNTs in the range of 30–450 °C. In the first stage, in the low-temperature range (30–150 °C), the weight loss of all samples is attributed to the removal of moisture and the dopant (Cl^-)—the process is similar to that observed for PANI/MWCNT composites [155]. According to the DTG data (Figure S3, curves 1, 2), the second stage corresponds to the temperature range of 150–260 °C and is associated with the removal of dopant residues and molecules of bound water [102,156]. In the third stage, in the temperature

https://doi.org/10.3390/polym18060000

range of 260–450 °C, the decomposition of the framework of polymer chains of varying degrees of polymerization is observed [102,157,158].

Table 3. Thermal properties.

SAMPLE	$T_{5\%}$, °C	$T_{50\%}$, °C	$T_{max \cdot dm/dt}$, °C	m_{res}, %
MWCNTs				99.8
PCPAB	110.7	307.9	286.2	44.7
PCPAB/ZnO	121.4	296.5	282.7	41.8
P/MWCNT-1	152.5	>450	283.5	53
P/MWCNT-2.1	185	436.7	296.7	49.3
P/MWCNT-2.2	177	>450	295	51.1
P/MWCNT-3.1	176	438.4	297	49.4
P/MWCNT-3.2	218.9	>450	298.4	52

m_{res}, %—residual mass of the sample at 450 °C; T_{max}—temperature at which the maximum change in mass occurred.

A slight increase in thermal stability in the temperature range up to 250 °C in the PCPAB/ZnO sample (Figure 14, curve 2) in comparison to PCPAB is associated with a lower dopant content [159]. The maximum weight loss rate for PCPAB/ZnO (Figure S3, curve 2) was observed at a lower temperature ($T_{max \cdot dm/dt}$ = 282.7 °C) than for PCPAB ($T_{max \cdot dm/dt}$ = 286.2 °C) (Table 3). This is due to a higher content of the low-molecular-weight fraction and a decrease in the degree of doping in the sample, which is consistent with the spectral analysis data in Section 3.1. The shoulder in the region of $T_{max \cdot dm/dt}$ = 306 °C and 299 °C for PCPAB and PCPAB/ZnO (Figure S3, curves 1, 2; Table 3), respectively, demonstrates a decrease in the decomposition rate of the composites due to the activation of crosslinking processes [102,160].

In all samples containing MWCNTs (Figure 14, curves 3–7), an increase in thermal stability relative to PCPAB was observed, since MWCNTs has a high temperature resistance (Figure 14, curve 0; Table 3). In P/MWCNT-1 m_{res} is 53%, while m_{res} (MWCNTs) is 99.8%, indicating the formation of a composite material and interaction between its components. This is also evidenced by the significant decrease in the weight loss rate shown by the DTG curve of P/MWCNT-1 (Figure S3, curve 3), which is explained by the fact that the MWCNTs are present in the sample in a pure form. During prolonged ultrasonic treatment of the nanotubes in the mixture of components during the preparation of P/MWCNT-1, they acquire a rougher and more defective surface, which enhances the interaction between the PCPAB macromolecules and the filler [161,162]. At the same time, a decrease in the temperature of the maximum weight loss rate for P/MWCNT-1 ($T_{max \cdot dm/dt}$ = 283.5 °C) compared to PCPAB indicates an increase in the content of the low-molecular-weight fraction in the polymer and a decrease in the amount of dopant as a result of ultrasonic treatment. The medium-intensity shoulder (295–315 °C) with a constrained maximum at ~306 °C on the DTG curve of P/MWCNT-1 indicates a slight increase in the thermal stability of the sample, which is associated with the presence of a certain number of cross-linked fragments in the structure of PCPAB formed under the influence of ultrasound treatment [102,163].

The TGA and DTG data (Figures 14 and S3, curves 4, 5) showed an increase in the thermal stability of the composites obtained via Method 2. $T_{max \cdot dm/dt}$ of the P/MWCNT-2.1 and P/MWCNT-2.2 samples shifted to the region of higher temperatures and is 296.7 °C and 295 °C (Table 3), respectively, which indicates a stronger interaction between the nanotubes and the polymer coating formed on their surface. The maximum mass loss rate of the samples (Figure S3, curves 4.5) is slightly higher than in P/MWCNT -1, since the

number of shorter PCPAB macromolecules increases during polymerization in the presence of nanotubes [122]. The slight decrease in $T_{max \cdot dm/dt}$ and increase in $T_{5\%}$ (Table 3) for P/MWCNT-2.2 is a consequence of an increase in the content of MWCNTs in the composite. This led to a decrease in the interchain interaction in PCPAB due to a violation of the regularity of packaging relative to each other of polymer chains formed in the volume of the reaction solution, and, as a result, an increase in the amount of incorporated moisture in PCPAB.

In P/MWCNT-3.1 and P/MWCNT-3.2, as opposed to P/MWCNT-2.1 and P/MWCNT-2.2, $T_{max \cdot dm/dt}$ and $T_{5\%}$ (Table 3) increase with increasing MWCNTs content. The $T_{5\%}$ parameter, which in our case relates to the temperature range of dopant removal and low molecular weight products [102], is lower for P/MWCNT-3.1 (177 °C) than for P/MWCNT-2.2 (185 °C). This is due to the use of ZnO, which lowers the pH of the reaction solution and increases the number of low molecular weight moieties in the polymer, as discussed in Section 3.1. However, compared to the samples obtained with Method 2, an increase in $T_{max \cdot dm/dt}$ was observed for P/MWCNT-3.1 (297.1 °C) and P/MWCNT-3.2 (298.4 °C), respectively, and a slight decrease in the rate of weight loss (Figure S3, Curves 6, 7; Table 3). Therefore, ZnO and its salts provided a denser packing of PCPAB chains around the nanotubes by increasing the effective specific surface area of the filler and enhancing the interaction between PCPAB and MWCNTs. This increases the thermal stability of the composite.

Thus, composite materials based on PCPAB and MWCNTs exhibited increased thermal stability compared to PCPAB. TGA and DTG methods showed that by adjusting the component ratios in the composite obtained using method 3, the thermal stability of the material can be improved.

4. Conclusions

For the first time, nanocomposites based on the conducting polymer PCPAB and MWCNTs were obtained by ultrasonic mixing of PCPAB and MWCNTs in an aqueous HCl solution, under conditions of in situ oxidative polymerization of CPAB in the presence of MWCNTs and under conditions of in situ oxidative polymerization of CPAB in the simultaneous presence of MWCNTs and ZnO.

It was established that the preparation conditions of hybrid nanocomposites significantly influence the formation of their chemical structure, as well as their functional properties, such as thermal stability and electrical conductivity.

Using FTIR and Raman spectroscopy, it was proven that the formation of hybrid nanocomposites occurs due to non-covalent bonding—π-stacking between the phenyl rings of PCPAB and the aromatic structures of MWCNTs.

It was shown that, for composites obtained by mixing PCPAB and MWCNTs, the adhesion of the polymer to the rough surface of ultrasonically treated nanotubes is of great importance.

Under conditions of in situ oxidative polymerization of CPAB in the presence of MWCNTs, the latter act as a template on which a polymer layer was formed. The introduction of ZnO particles into the reaction solution leads to the formation of a more ordered and dense polymer coating on the surface of the nanotubes. This is explained by the fact that, in addition to Van der Waals interactions between PCPAB chains, interchain complexation occurred involving Zn^{2+} ions formed in the acidic reaction medium. In this case, complexation occurred both between the main and side polymer chains.

Similar to PCPAB in its salt form, all obtained nanocomposites are electrically conductive. The electrical conductivity values are determined by the preparation conditions of the nanocomposites and reach 1.1 S·cm^{-1}, which is more than an order of magnitude higher

https://doi.org/10.3390/polym18060000

than the conductivity of the pristine polymer. Frequency dependencies of conductivity indicate a hopping mechanism of charge carrier transport in both the pristine polymer and the nanocomposites. Thermal degradation of the nanocomposites in the range of 30–450 °C includes dedoping processes, moisture removal, and the removal of low-molecular-weight polymerization products. Nanocomposites obtained in the presence of ZnO exhibit better thermal stability.

A subsequent development of our work will involve the fabrication of hybrid electrodes for supercapacitors, featuring an electroactive layer based on PCPAB/MWCNTs obtained under optimal conditions, and the study of their electrochemical behavior.

Supplementary Materials: The following supporting information can be downloaded at https://www.mdpi.com/article/10.3390/polym18060000/s1, Table S1. Relative intensities of the δCCH bands of 1,4-N- and mono-N-substituted aromatic rings in the spectra of PCPAB in its neutral form.; Figure S1. IR spectra of samples (salt form) obtained via oxidative polymerization of CPAB in HCl: PCPAB (1), P/MWCNT-1 (2); Figure S2. PCPAB polymer chain growth along the surface of the nanotube (based on the mechanism of oxidative polymerization of CPAB presented in [99,100]); Figure S3. DTG curves—PCPAB (1); PCPAB/ZnO (2); P/MWCNT-1 (3); P/MWCNT-2.1 (4); P/MWCNT-2.2 (5); P/MWCNT-3.1 (6); P/MWCNT-3.2 (7).

Author Contributions: Conceptualization, S.G.K. and G.P.K.; methodology, S.G.K.; formal analysis, G.N.B. and S.G.K.; synthesis, S.G.K.; investigation, S.G.K., G.N.B., D.G.M., V.V.K. and A.A.V.; writing—original draft preparation, S.G.K. and G.N.B.; supervision, writing—review and editing, G.P.K. All authors have read and agreed to the published version of the manuscript.

Funding: This research received no external funding.

Institutional Review Board Statement: Not applicable.

Data Availability Statement: The original contributions presented in this study are included in the article/Supplementary Material. Further inquiries can be directed to the corresponding author.

Acknowledgments: This work was carried out within the State Program of TIPS RAS. This work was performed using the equipment of the Shared Research Center "Analytical center of deep oil processing and petrochemistry of TIPS RAS".

Conflicts of Interest: The authors declare no conflicts of interest.

References

1. Baughman, R.H.; Zakhidov, A.A.; De Heer, W.A. Carbon nanotubes—The route toward applications. *Science* **2002**, *297*, 787–792. [CrossRef]
2. Li, H.; Xu, C.; Srivastava, N.; Banerjee, K. Carbon nanomaterials for next-generation interconnects and passives: Physics, status, and prospects. *IEEE Trans. Electron Devices* **2009**, *56*, 1799–1821. [CrossRef]
3. Zaporotskova, I.V.; Boroznina, N.P.; Parkhomenko, Y.N.; Kozhitov, L.V. Carbon nanotubes: Sensor properties. A review. *Modern Electronic Materials. Mod. Electron. Mater.* **2016**, *2*, 95–105. [CrossRef]
4. Oueiny, C.; Berlioz, S.; Perrin, F.-X. Carbon nanotube–polyaniline composites. *Prog. Polym. Sci.* **2014**, *39*, 707–748. [CrossRef]
5. Anzar, N.; Hasan, R.; Tyagi, M.; Yadav, N.; Narang, J. Carbon nanotube—A review on Synthesis, Properties and plethora of applications in the field of biomedical science. *Sens. Int.* **2020**, *1*, 100003. [CrossRef]
6. Güney, S. Electrosynthesis of Molecularly Imprinted Poly-o-phenylenediamine on MWCNT Modified Electrode for Selective Determination of Meldonium. *Electroanalysis* **2019**, *31*, 661–670. [CrossRef]
7. Pisarevskaya, E.Y.; Ozkan, S.Z.; Petrov, V.A.; Efimov, O.; Karpacheva, G.P. New electrodes based on composites of polyphenothiazine with carbon nanomaterials. *Mater. Chem. Phys.* **2024**, *312*, 128668. [CrossRef]
8. Jariwala, D.; Sangwan, V.K.; Lauhon, L.J.; Marks, T.J.; Hersam, M.C. Carbon nanomaterials for electronics, optoelectronics, photovoltaics, and sensing. *Chem. Soc. Rev.* **2013**, *42*, 2824–2860. [CrossRef]
9. Fan, W.; Zhang, Y.; Guo, C.-Y.; Chen, G. Toward high thermoelectric performance for polypyrrole composites by dynamic 3-phase interfacial electropolymerization and chemical doping of carbon nanotubes. *Compos. Sci. Technol.* **2019**, *183*, 107794. [CrossRef]
10. Xue, Y.; Gao, C.; Liang, L.; Wang, X.; Chen, G. Nanostructure controlled construction of high-performance thermoelectric materials of polymers and their composites. *J. Mater. Chem. A* **2018**, *6*, 22381–22390. [CrossRef]

11. Mathew, R.J.; Chandran, A.R.; Syamkishor, K.S.; Joseph, L.K.; Saji, K.J.; Sajeev, U.S. Tunable nonlinear optical properties in polyaniline-multiwalled carbon nanotube (PANI-MWCNT) system probed under pulsed Nd:YAG laser. *Next Mater.* **2025**, *6*, 100431. [CrossRef]
12. Lehman, J.H.; Terrones, M.; Mansfield, E.; Hurst, K.E.; Meunier, V. Evaluating the characteristics of multiwall carbon nanotubes. *Carbon* **2011**, *49*, 2581–2602. [CrossRef]
13. Ansari, S.A.; Parveen, N.; Ansari, M.Z.; Alsulaim, G.M.; Alam, M.W.; Khan, M.Y.; Umar, A.; Hussain, I.; Zhang, K. Exploring recent advances in the versatility and efficiency of carbon materials for next generation supercapacitor applications: A comprehensive review. *Prog. Mater. Sci.* **2025**, *154*, 101493. [CrossRef]
14. Lawal, A.T. Recent application of carbon nanotubes in energy storage and conversion devices. *Carbon Trends* **2025**, *19*, 100470. [CrossRef]
15. Prasannakumar, A.T.; Rohith, R.; Manju, V.; Mohan, R.R.; Varma, S.J. High energy density electrodes based on solution intercalated, self-stabilized dispersion polymerized polyaniline/MWCNT hybrids for supercapacitors. *J. Energy Storage* **2024**, *76*, 109732. [CrossRef]
16. Sun, Y.; Wilson, S.R.; Schuster, D.I. High Dissolution and Strong Light Emission of Carbon Nanotubes in Aromatic Amine Solvents. *J. Am. Chem. Soc.* **2001**, *123*, 5348–5349. [CrossRef]
17. Rajput, S.; Panwar, A.K.; Gupta, A. Study of lithium diffusion properties and electrochemical performance of SnSe/C and SnSe/MWCNT composite anode for Li-ion batteries. *Solid State Ion.* **2023**, *394*, 116206. [CrossRef]
18. Gandara, M.; Gonçalves, E.S. Polyaniline supercapacitor electrode and carbon fiber graphene oxide: Electroactive properties at the charging limit. *Electrochim. Acta* **2020**, *345*, 136197. [CrossRef]
19. Ha, J.-S.; Lee, J.-M.; Lee, H.-R.; Huh, P.; Jo, N.-J. Polymer nanocomposite electrode consisting of polyaniline and modified multi-walled carbon nanotube for rechargeable battery. *J. Nanosci. Nanotechnol.* **2015**, *15*, 8977–8983. [CrossRef]
20. Manimekalai, A.; Velu, K.S.; Mohandoss, S.; Ahma, R.W.N.; Seo, J.H.; Sun, S.; Lee, Y.R. Poly(aniline)-multiwall carbon nanotube (PANI@MWCNT) composite as high-cost Pt free counter electrode for dye-sensitized solar cells. *Curr. Appl. Phys.* **2024**, *68*, 230–237. [CrossRef]
21. Gao, Y.; He, S.; Hu, J.; Wu, C.; Chen, Z.; Cen, H. A simple strategy to significantly improve the anticorrosion and aging resistance of epoxy coatings by adding polyaniline modified multi-walled carbon nanotubes. *Polym. Degrad. Stab.* **2024**, *230*, 111027. [CrossRef]
22. Wu, Z.; Yao, X.; Xing, Y.; Huang, P.; Li, B.; Liu, L. Enhanced electromagnetic shielding and surface modulation of electrospun cellulose acetate-based fiber membranes via multi-walled carbon nanotubes and in-situ polyaniline polymerization. *Colloids Surf. A Physicochem. Eng. Asp.* **2025**, *718*, 136957. [CrossRef]
23. Kaushik, P.; Bharti, R.; Sharma, R.; Verma, M.; Olsson, R.T.; Pandey, A. Progress in synthesis and applications of Polyaniline-Coated Nanocomposites: A comprehensive review. *Eur. Polym. J.* **2024**, *221*, 113574. [CrossRef]
24. Stejskal, J. Polymers of phenylenediamines. *Prog. Polym. Sci.* **2015**, *41*, 1–31. [CrossRef]
25. Jadoun, S.; Riaz, U.; Yáñez, J.; Chauhan, N.P.S. Synthesis, characterization and potential applications of Poly(o-phenylenediamine) based copolymers and Nanocomposites: A comprehensive review. *Eur. Polym. J.* **2021**, *156*, 110600. [CrossRef]
26. Cataldo, F.; Maltese, P. Synthesis of alkyl and N-alkyl-substituted polyanilines: A study on their spectral properties and thermal stability. *Eur. Polym. J.* **2002**, *38*, 1791–1803. [CrossRef]
27. Manohar, S.K.; MacDiarmid, A.G.; Cromack, K.R.; Ginder, J.M.; Epstein, A.J. N-substituted derivatives of polyaniline. *Synth. Met.* **1989**, *29*, 349–356. [CrossRef]
28. Bhadra, S.; Singha, N.K.; Khastgir, D. Effect of aromatic substitution in aniline on the properties of polyaniline. *Eur. Polym. J.* **2008**, *44*, 1763–1770. [CrossRef]
29. Zhang, Y.; Duan, Y.; Liu, J.; Ma, G.; Huang, M. Wormlike Acid-Doped Polyaniline: Controllable Electrical Properties and Theoretical Investigation. *J. Phys. Chem. C* **2018**, *122*, 2032–2040. [CrossRef]
30. Lin, K.-Y.; Hu, L.-W.; Chen, K.-L.; Siao, M.-D.; Ji, W.-F.; Yang, C.-C.; Yeh, J.-M.; Chiu, K.-C. Characterization of polyaniline synthesized from chemical oxidative polymerization at various polymerization temperatures. *Eur. Polym. J.* **2017**, *88*, 311–319. [CrossRef]
31. Bhadra, S.; Khastgir, D.; Singha, N.K.; Lee, J.H. Progress in preparation, processing and applications of polyaniline. *Prog. Polym. Sci.* **2009**, *34*, 783–810. [CrossRef]
32. Palaniappan, S.; John, A. Polyaniline materials by emulsion polymerization pathway. *Prog. Polym. Sci.* **2008**, *33*, 732–758. [CrossRef]
33. Alabdullah, S.S.M.; Ismail, H.K.; Ryder, K.S.; Abbott, A.P. Evidence supporting an emulsion polymerisation mechanism for the formation of polyaniline. *Electrochim. Acta* **2020**, *354*, 136737. [CrossRef]
34. Li, J.; Cui, M.; Lai, Y.; Zhang, Z.; Lu, H.; Fang, J.; Liu, Y. Investigation of polyaniline co-doped with Zn2+ and H+ as the electrode material for electrochemical supercapacitors. *Synth. Met.* **2010**, *160*, 1228–1233. [CrossRef]

35. Ghosh, D.; Giri, S.; Mandal, A.; Das, C.K. Supercapacitor based on H^+ and Ni^{2+} co-doped polyaniline–MWCNTs nanocomposite: Synthesis and electrochemical characterization. *RSC Adv.* **2013**, *3*, 11676. [CrossRef]
36. Zhang, H.; Yuan, J.; Liu, Y.; Zheng, R.; Jia, C. Zn^{2+} assisted co-construction of polyaniline electrochromic device with enhanced infrared modulation. *Electrochim. Acta* **2025**, *529*, 146354. [CrossRef]
37. Iijima, S. Helical microtubules of graphitic carbon. *Nature* **1991**, *354*, 56–58. [CrossRef]
38. Hughes, K.J.; Iyer, K.A.; Bird, R.E.; Ivanov, J.; Banerjee, S.; Georges, G.; Zhou, Q.A. Review of Carbon Nanotube Research and Development: Materialsand Emerging Applications. *ACS Appl. Nano Mater.* **2024**, *7*, 18695–18713. [CrossRef]
39. Gajendran, P.; Saraswathi, R. Polyaniline-carbon nanotube composites. *Pure Appl. Chem.* **2008**, *80*, 2377–2395. [CrossRef]
40. Guo, M.; Chen, J.; Li, J.; Tao, B.; Yao, S. Fabrication of polyaniline/carbon nanotube composite modified electrode and its electrocatalytic property to the reduction of nitrite. *Anal. Chim. Acta* **2005**, *532*, 71–77. [CrossRef]
41. Tan, W.; Stallard, J.C.; Jo, C.; De Volder, M.F.L.; Fleck, N.A. The mechanical and electrochemical properties of polyaniline-coated carbon nanotube mat. *J. Energy Storage* **2021**, *41*, 102757. [CrossRef]
42. Okotrub, A.V.; Asanov, I.P.; Galkin, P.S.; Bulusheva, L.G.; Chekhova, G.N.; Kurenya, A.G.; Shubin, Y.V. Composites Based on Polyaniline and Aligned Carbon Nanotubes. *Polym. Sci. Ser. B* **2010**, *52*, 101–108. [CrossRef]
43. Wu, R.; Yuan, H.; Liu, C.; Lan, J.-L.; Yang, X.; Lin, Y.-H. Flexible PANI/SWCNT thermoelectric films with ultrahigh electrical conductivity. *RSC Adv.* **2018**, *8*, 26011–26019. [CrossRef] [PubMed]
44. Feng, L.; Wu, R.; Liu, C.; Lan, J.; Lin, Y.-H.; Yang, X. Facile Green Vacuum-Assisted Method for Polyaniline/SWCNT Hybrid Films with Enhanced Thermoelectric Performance by Interfacial Morphology Control. *ACS Appl. Energy Mater.* **2021**, *4*, 4081–4089. [CrossRef]
45. Nagaraja, K.K.; Pramodini, S.; Poornesh, P.; Telenkov, M.P.; Kityk, I.V. Nonlinear optical properties of polyaniline and poly(o-toluidine) composite thin films with multi walled carbon nano tubes. *Phys. B Condens. Matter* **2017**, *512*, 45–53. [CrossRef]
46. Joseph, L.; Flora, J.R.V.; Park, Y.-G.; Badawy, M.; Saleh, H.; Yoon, Y. Removal of natural organic matter from potential drinking water sources by combined coagulation and adsorption using carbon nanomaterials. *Sep. Purif. Technol.* **2012**, *95*, 64–72. [CrossRef]
47. Yang, K.; Xing, B. Adsorption of fulvic acid by carbon nanotubes from water. *Environ. Pollut.* **2009**, *157*, 1095–1100. [CrossRef]
48. Konyushenko, E.N.; Stejskal, J.; Trchova, M.; Hradil, J.; Kovářová, J.; Prokeš, J.; Cieslar, M.; Hwang, J.Y.; Chen, K.H.; Sapurina, I. Multi-wall carbon nanotubes coated with polyaniline. *Polymer* **2006**, *47*, 5715–5723. [CrossRef]
49. Cochet, M.; Maser, W.K.; Benito, A.M.; Callejas, M.A.; Martínez, M.T.; Benoit, J.-M.; Schreiber, J.; Chauvet, O. Synthesis of a new polyaniline/nanotube composite: "in-situ" polymerization and charge transfer through site-selective interaction. *Chem. Commun.* **2001**, *16*, 1450–1451. [CrossRef]
50. Oraon, R.; De Adhikari, A.; Tiwari, S.K.; Nayak, G.C. Enhanced Specific Capacitance of Self-Assembled Three-Dimensional Carbon Nanotube/Layered Silicate/Polyaniline Hybrid Sandwiched Nanocomposite for Supercapacitor Applications. *ACS Sustain. Chem. Eng.* **2016**, *4*, 1392–1403. [CrossRef]
51. Kim, J.-W.; Siochi, E.J.; Carpena-Núñez, J.; Wise, K.E.; Connell, J.W.; Lin, Y.; Wincheski Russell, A. Polyaniline/Carbon Nanotube Sheet Nanocomposites: Fabrication and Characterization. *ACS Appl. Mater. Interfaces* **2013**, *5*, 8597–8606. [CrossRef] [PubMed]
52. Roy, A.; Ray, A.; Saha, S.; Das, S. Investigation on energy storage and conversion properties of multifunctional PANI-MWCNT composite. *Int. J. Hydrogen Energy* **2018**, *43*, 7128–7139. [CrossRef]
53. Salvatierra, R.V.; Zitzer, G.; Savu, S.-A.; Alves, A.P.; Zarbin, A.J.G.; Chassé, T.; Casu, M.B.; Rocco, M.L.M. Carbon nanotube/polyaniline nanocomposites: Electronic structure, doping level and morphology investigations. *Synth. Met.* **2015**, *203*, 16–21. [CrossRef]
54. Yao, Q.; Chen, L.; Zhang, W.; Liufu, S.; Chen, X. Enhanced Thermoelectric Performance of Single-Walled Carbon Nanotubes/Polyaniline Hybrid Nanocomposites. *ACS Nano* **2010**, *4*, 2445–2451. [CrossRef]
55. Ogurtsov, N.A.; Noskov, Y.V.; Bliznyuk, V.N.; Ilyin, V.G.; Wojkiewicz, J.-L.; Fedorenko, E.A.; Pud, A.A. Evolution and Interdependence of Structure and Properties of Nanocomposites of Multiwall Carbon Nanotubes with Polyaniline. *J. Phys. Chem. C* **2015**, *120*, 230–242. [CrossRef]
56. Deb, K.; Sarkar, K.; Bera, A.; Debnath, A.; Sah, B. Coupled polaron-electron charge transport in graphite functionalized polyaniline on cellulose: Metal free flexible p-type semiconductor. *Synth. Met.* **2018**, *245*, 96–101. [CrossRef]
57. Ginic-Markovic, M.; Matisons, J.G.; Cervini, R.; Simon, G.P.; Fredericks, P.M. Synthesis of New Polyaniline-Nanotube Composites Using Ultrasonically Initiated Emulsion Polymerization. *Chem. Mater.* **2006**, *18*, 6258–6265. [CrossRef]
58. Ardani, M.R.; Pang, A.; Pal, U.; Zheng, R.; Arsad, A.; Hamzah, A.A.; Ahmadipour, M. Ultrasonic-assisted polyaniline-multiwall carbon nanotube photocatalyst for efficient photodegradation of organic pollutants. *J. Water Process Eng.* **2022**, *46*, 102557. [CrossRef]
59. Suckeveriene, R.Y.; Zelikman, E.; Mechrez, G.; Tzur, A.; Frisman, I.; Cohen, Y.; Narkis, M. Synthesis of Hybrid Polyaniline-Carbon Nanotube Nanocomposites by Dynamic Interfacial Inverse Emulsion Polymerization Under Sonication. *J. Appl. Polym. Sci.* **2011**, *120*, 676–682. [CrossRef]

60. Zelikman, E.; Suckeveriene, R.Y.; Mechrez, G.; Narkis, M. Fabrication of composite polyaniline/CNT nanofibers using an ultrasonically assisted dynamic inverse emulsion polymerization technique. *Polym. Adv. Technol.* **2010**, *21*, 150–152. [CrossRef]
61. Liu, X.; Sun, Y.; Tong, Y.; Wang, X.; Zheng, J.; Wu, Y.; Li, H.; Niu, L.; Hou, Y. Exploration in materials, electrolytes and performance towards metal ion (Li, Na, K, Zn and Mg)-based hybrid capacitors: A review. *Nano Energy* **2021**, *86*, 106070. [CrossRef]
62. Kim, S.W.; Kim, T.; Kim, Y.S.; Choi, H.S.; Lim, H.J.; Yang, S.J.; Park, C.R. Surface modifications for the effective dispersion of carbon nanotubes in solvents and polymers. *Carbon* **2012**, *50*, 3–33. [CrossRef]
63. Wei, K.; Tang, H.; Qu, G.; Pan, K. Selective detection of hydroxyl radicals by molecularly imprinted electrochemical sensors constructed with MWCNTs-COOH-modified electrodes. *Microchem. J.* **2024**, *205*, 111194. [CrossRef]
64. Naqvi, S.T.R.; Rasheed, T.; Hussain, D.; Ha, M.N.; Majeed, S.; Shafi, S.; Ahmed, N.; Nawaz, R. Modification strategies for improving the solubility/dispersion of carbon nanotubes. *J. Mol. Liq.* **2020**, *297*, 111919. [CrossRef]
65. Teoh, W.-C.; Yeoh, W.-M.; Mohamed, A.R. Evaluation of Different Oxidizing Agents on Effective Covalent Functionalization of Multiwalled Carbon Nanotubes. *Fuller. Nanotub. Carbon Nanostructures* **2018**, *26*, 846–850. [CrossRef]
66. Jun, L.Y.; Mubarak, N.M.; Yon, L.S.; Bing, C.H.; Khalid, M.; Abdullah, E.C. Comparative study of acid functionalization of carbon nanotube via ultrasonic and reflux mechanism. *J. Environ. Chem. Eng.* **2018**, *6*, 5889–5896. [CrossRef]
67. Osorio, G.; Silveira, I.C.L.; Bueno, V.L.; Bergmann, C.P. H_2SO_4/HNO_3/HCl–Functionalization and its Effect on Dispersion of Carbon Nanotubes in Aqueous Media. *Appl. Surf. Sci. Part 1* **2008**, *255*, 2485–2489. [CrossRef]
68. Hernadi, K.; Siska, A.; Thiên-Nga, L.; Forró, L.; Kiricsi, I. Reactivity of Different Kinds of Carbon During Oxidative Purification of Catalytically Prepared Carbon Nanotubes. *Solid State Ion.* **2001**, *141–142*, 203–209. [CrossRef]
69. Wang, M.W.; Wang, J.; Qu, J.W. Study on the Chemical Modification of the Walls of Carbon Nanotubes by $K_2Cr_2O_7$and HNO_3. *Adv. Mater. Res.* **2011**, *197–198*, 571–574. [CrossRef]
70. Huang, J.-Q.; Zhang, Q.; Zhao, M.-Q.; Wei, F. The Release of Free Standing Vertically aligned Carbon Nanotube Arrays from a Substrate Using CO2 Oxidation. *Carbon* **2010**, *48*, 1441–1450. [CrossRef]
71. Ran, M.; Sun, W.; Liu, Y.; Chu, W.; Jiang, C. Functionalization of Multi-walled Carbon Nanotubes Using Water-assisted Chemical Vapor Deposition. *J. Solid State Chem.* **2013**, *197*, 517–522. [CrossRef]
72. Solhy, A.; Machado, B.F.; Beausoleil, J.; Kihn, Y.; Gonçalves, F.; Pereira, M.; Órfão, J.; Figueiredo, J.; Serp, P. MWCNT Activation and its Influence on the Catalytic Performance of Pt/MWCNT Catalysts for Selective Hydrogenation. *Carbon* **2008**, *46*, 1194–1207. [CrossRef]
73. Chen, L.; Xie, H. Surfactant-free nano fluids containing double- and single-walled carbon nanotubes functionalized by a wet-mechanochemical reaction. *Thermochim. Acta* **2010**, *497*, 67–71. [CrossRef]
74. Basiuk, E.V.; Basiuk, V.A.; Banuelos, J.-G.; Saniger-Blesa, J.-M.; Pokrovskiy, V.A.; Gromovoy, T.Y.; Mischanchuk, A.V.; Mischanchuk, B.G. Interaction of Oxidized Single-walled Carbon Nanotubes with Vaporous Aliphatic Amines. *J. Phys. Chem. B* **2002**, *10*, 1588–1597. [CrossRef]
75. Choudhury, A.; Kar, P. Doping effect of carboxylic acid group functionalized multi-walled carbon nanotube on polyaniline. *Compos. Part. B Eng.* **2011**, *42*, 1641–1647. [CrossRef]
76. Xu, J.; Yao, P.; Li, X.; He, F. Synthesis and characterization of water-soluble and conducting sulfonated polyaniline/para-phenylenediamine-functionalized multi-walled carbon nanotubes nanocomposite. *Mater. Sci. Eng. B* **2008**, *151*, 210–219. [CrossRef]
77. Ziegler, K.J.; Gu, Z.; Peng, H.; Flor, E.L.; Hauge, R.H.; Smalley, R.E. Controlled Oxidative Cutting of Single-Walled Carbon Nanotubes. *J. Am. Chem. Soc.* **2005**, *127*, 1541–1547. [CrossRef]
78. Worsley, K.A.; Kalinina, I.; Bekyarova, E.; Haddon, R.C. Functionalization and Dissolution of Nitric Acid Treated Singlewalled Carbon Nanotubes. *J. Am. Chem. Soc.* **2009**, *131*, 18153–18158. [CrossRef]
79. Sezer, N.; Koç, M. Oxidative acid treatment of carbon nanotubes. *Surf. Interfaces* **2019**, *14*, 1–8. [CrossRef]
80. Bumika, M.; Mallick, M.K.; Mohanty, S.; Nayak, S.K.; Palai, A.K. One-pot electrodeposition of polyaniline/SWCNT/ZnO film and its positive influence on photovoltaic performance as counter electrode material. *Mater. Lett.* **2020**, *279*, 128473. [CrossRef]
81. Amenu, B.; Taddesse, A.M.; Kebede, T.; Mengesha, E.T.; Bezu, Z. Polyaniline-supported MWCNTs/ZnO/Ag_2CO_3 composite with enhanced photocatalytic and antimicrobial applications. *Environ. Nanotechnol. Monit. Manag.* **2024**, *21*, 100926. [CrossRef]
82. Khan, A.A.; Khalid, M. Synthesis of nano-sized ZnO and polyaniline-zinc oxide composite: Characterization, stability in terms of DC electrical conductivity retention and application in ammonia vapor detection. *J. Appl. Polym. Sci.* **2010**, *117*, 1601–1607. [CrossRef]
83. Sharma, B.K.; Gupta, A.K.; Kharea, N.; Dhawan, S.K.; Gupta, H.C. Synthesis and characterization of polyaniline–ZnO composite and its dielectric behavior. *Synth. Met.* **2009**, *159*, 391–395. [CrossRef]
84. Jalali, H.; Eslami-Farsani, R.; Ramezanzadeh, B. Zinc-doped sulfonated polyaniline tailored Multi-walled carbon nanotubes (Zn-SPANI-MWCNT); A novel nano-hybrid for designing a smart self-healable epoxy coating. *J. Ind. Eng. Chem.* **2023**, *122*, 397–414. [CrossRef]

https://doi.org/10.3390/polym18060000

85. Najmi, P.; Keshmiri, N.; Ramezanzadeh, M.; Ramezanzadeh, B. Highly improving the mechanical-responses/thermal-stability of the epoxy nano-composite using novel highly-oxidized multi-walled carbon nanotubes (OMWCNT) functionalized by Zinc-doped Polyaniline (PANI) nanofibers. *J. Taiwan Inst. Chem. Eng.* **2021**, *119*, 245–258. [CrossRef]
86. Ong, P.J.; Lee, H.Y.S.; Wang, S.; Thitsartarn, W.; Zhang, X.; Kong, J.; Kai, D.; Tan, B.H.; Wang, P.; Qu, Z.; et al. Recent advances in enhanced thermal property in phase change materials using carbon nanotubes: A review. *Sol. Energy Mater. Sol. Cells* **2025**, *279*, 113228. [CrossRef]
87. Lavagna, L.; Nistico, R.; Musso, S.; Pavese, M. Functionalization as a way to enhance dispersion of carbon nanotubes in matrices: A review. *Mater. Today Chem.* **2021**, *20*, 100477. [CrossRef]
88. Zhou, Y.; Fang, Y.; Ramasamy, R.P. Non-Covalent Functionalization of Carbon Nanotubes for Electrochemical Biosensor Development. *Sensors* **2019**, *19*, 392. [CrossRef]
89. Hwang, J.-Y.; Nish, A.; Doig, J.; Douven, S.; Chen, C.-W.; Chen, L.-C.; Nicholas, R.J. Polymer Structure and Solvent Effects on the Selective Dispersion of Single-Walled Carbon Nanotubes. *J. Am. Chem. Soc.* **2008**, *130*, 3543–3553. [CrossRef]
90. Yu, J.; Grossiord, N.; Koning, C.E.; Loos, J. Controlling the dispersion of multi-wall carbon nanotubes in aqueous surfactant solution. *Carbon* **2007**, *45*, 618–623. [CrossRef]
91. Star, A.; Liu, Y.; Grant, K.; Ridvan, L.; Stoddart, J.F.; Steuerman, D.W.; Boukai, M.R.; Heath, J.R. Noncovalent side-wall functionalization of single-walled carbon nanotubes. *Macromolecules* **2003**, *36*, 553–560. [CrossRef]
92. Panczyk, T.; Wolski, P.; Jagusiak, A.; Drach, M. Molecular dynamics study of Congo red interaction with carbon nanotubes. *RSC Adv.* **2014**, *4*, 47304–47312. [CrossRef]
93. Wu, T.-M.; Chang, H.-L.; Lin, Y.-W. Synthesis and characterization of conductive polypyrrole/multi-walled carbon nanotubes composites with improved solubility and conductivity. *Compos. Sci. Technol.* **2009**, *69*, 639–644. [CrossRef]
94. Hekmat, F.; Shahrokhian, S.; Mirzaei, Y. Effect of Long-Chain Ionic Liquids on the Capacitive Performance of Carbon Nanotube-Sulfonated Polyaniline Hydrogels for Energy Storage Applications. *J. Phys. Chem. C* **2020**, *124*, 9810–9821. [CrossRef]
95. Ginder, J.M.; Epstein, A.J.; MacDiarmid, A.G. Phenyl ring rotations, structural order and electronic states in polyaniline. *Synth. Met.* **1990**, *37*, 45–55. [CrossRef]
96. MacDiarmid, A.G.; Epstein, A.J. The concept of secondary doping as applied to polyaniline. *Synth. Met.* **1994**, *65*, 103–116. [CrossRef]
97. Umeyama, T.; Kawabata, K.; Tezuka, N.; Matano, Y.; Miyato, Y.; Matsushige, K.; Tsujimoto, M.; Isoda, S.; Takano, M.; Imahor, H. Dispersion of carbon nanotubes by photo- and thermal-responsive polymers containing azobenzene unit in the backbone. *Chem. Commun.* **2010**, *46*, 5969–5971. [CrossRef]
98. Bilalis, P.; Katsigiannopoulos, D.; Avgeropoulos, A.; Sakellariou, G. Non-covalent functionalization of carbon nanotubes with polymers. *RSC Adv.* **2014**, *4*, 2911–2934. [CrossRef]
99. Orlov, A.V.; Kiseleva, S.G.; Karpacheva, G.P.; Muratov, D.G. Peculiarities of Oxidative Polymerization of Diarylaminodichlorobenzoquinones. *Polymers* **2021**, *13*, 3657. [CrossRef]
100. Kiseleva, S.G.; Orlov, A.V.; Bondarenko, G.N.; Karpacheva, G.P. Oxidative polymerization of 3,6-dianiline-2,5-dichlorbenzoquinone and its copolymerization with aniline. *Polym. Sci. Ser. B* **2018**, *60*, 717–726. [CrossRef]
101. Abalyaeva, V.V.; Nikolaeva, G.V.; Kabachkov, E.N.; Kiseleva, S.G.; Orlov, A.V.; Efimov, O.N.; Karpacheva, G.P. Preparation and comparative study of electrochemical behavior of composite electrodes based on polyaniline and its N-substituted. *Polym. Sci.* **2018**, *60*, 511–520. [CrossRef]
102. Kiseleva, S.G.; Bondarenko, G.N.; Orlov, A.V.; Muratov, D.G.; Kozlov, V.V.; Vasilev, A.A.; Karpacheva, G.P. Hybrid Nanocomposites Based on Poly(3,6-dianiline-2,5-dichloro-1,4-benzoquinone): Synthesis, Structure and Properties. *Polymers* **2024**, *16*, 1832. [CrossRef] [PubMed]
103. Kondratyuk, P.; Yates, J.T., Jr. Molecular Views of Physical Adsorption Inside and Outside of Single-Wall Carbon. *Nanotubes. Acc. Chem. Res.* **2007**, *40*, 995–1004. [CrossRef] [PubMed]
104. Fu, F.; Wang, Q. Removal of heavy metal ions from wastewaters: A review. *J. Environ. Manag.* **2011**, *92*, 407–418. [CrossRef]
105. Han, J.; Dai, J.; Guo, R. Highly efficient adsorbents of poly(o-phenylenediamine) solid and hollow sub-microspheres towards lead ions: A comparative study. *J. Colloid Interface Sci.* **2011**, *356*, 749–756. [CrossRef]
106. Bhadra, S.; Khastgir, D. Determination of crystal structure of polyaniline and substituted polyanilines through powder X-ray diffraction analysis. *Polym. Test.* **2008**, *27*, 851–857. [CrossRef]
107. Stejskal, J.; Riede, A.; Hlavata, D.; Prokes, J.; Helmstedt, M.; Holler, P. The effect of polymerization temperature on molecular weight, crystallinity, and electrical conductivity of polyaniline. *Synth. Met.* **1998**, *96*, 55–61. [CrossRef]
108. Pouget, J.P.; Jozefowicz, M.E.; Epstein, A.J.; Tang, X.; MacDiarmid, A.G. X-ray Structure of Polyaniline. *Macromolecules* **1991**, *4*, 779–789. [CrossRef]
109. Palaniappan, I.S. Benzoyl peroxide oxidation route to polyaniline salts—Part I. *Polym. Adv. Technol.* **2004**, *15*, 111–117. [CrossRef]
110. Kim, B.-J.; Oh, S.-G.; Han, M.-G.; Im, S.-S. Synthesis and characterization of polyaniline nanoparticles in SDS micellar solutions. *Synth. Met.* **2001**, *122*, 297–304. [CrossRef]

111. Rafiq, S.; Lovely, M.A.; Mim, S.R.; Islam, M.S.; Hasan, M.; Billah, M.M. Structure controlled enhanced photocatalytic activity of polyaniline (PANI). *Heliyon* **2025**, *11*, 42888. [CrossRef] [PubMed]
112. Gospodinova, N.; Terlemezyan, L. Conducting polymers prepared by oxidative polymerization: Polyaniline. *Prog. Polym. Sci.* **1998**, *23*, 1443–1484. [CrossRef]
113. Orlov, A.V.; Kiseleva, S.G.; Yurchenko, O.Y.; Karpacheva, G.P. Oxidative polymerization of polyaniline in the presence of additional substrate. *Polym. Sci. Part A* **2000**, *42*, 1292–1297.
114. Mathew, R.J.; Radhakrishnan, P.; Pragash, R.; John, J.; Kumar, K.V.A.; Sajeev, U.S. The synergistic impact of multi walled carbon nanotubes on microstructural, morphological, and optical properties of polyaniline films. *ChemistrySelect* **2024**, *9*, 2023042102. [CrossRef]
115. Sharma, A.K.; Jain, P.K.; Vyas, R.; Mathur, V.; Jain, V.K. Study of thermal stability and dielectric behavior of PANI/MWCNT nanocomposite. *Mater. Today Proc.* **2021**, *38*, 1259–1262. [CrossRef]
116. Zou, L.; Lan, C.; Yang, L.; Xu, Z.; Chu, C.; Liu, Y.; Qiu, Y. The optimization of nanocomposite coating with polyaniline coated carbon nanotubes on fabrics for exceptional electromagnetic interference shielding. *Diam. Relat. Mater.* **2020**, *104*, 107757. [CrossRef]
117. Sreevidya, U.; Shalini, V.; Kavirajan, S.; Maiyelvaganan, K.R.; Prakash, M.; Bharathi, K.K.; Kumar, E.S.; Archana, J.; Harish, S.; Navaneethan, M. Investigation of non-covalent interactions in Polypyrrole/Polyaniline/Carbon black ternary complex for enhanced thermoelectric properties via interfacial carrier scattering and p-p stacking. *J. Colloid Interface Sci.* **2023**, *630*, 46–60. [CrossRef]
118. Ma, J.; Nan, X.; Liu, J.; Zhu, W.; Qin, W. Dispersion of pristine and polyaniline functionalized carbon nanotubes in designed solvent mixtures by Hansen solubility parameters. *Mater. Today Commun.* **2018**, *14*, 99–105. [CrossRef]
119. Baibarac, M.; Baltog, I.; Lefrant, S.; Mevellec, J.Y.; Chauvet, O. Polyaniline and Carbon Nanotubes Based Composites Containing Whole Units and Fragments of Nanotubes. *Chem. Mater.* **2003**, *15*, 4149–4156. [CrossRef]
120. Sukhananazerin, A.; Thalakkotur, L.M.; Biji, P. Highly sensitive, room temperature gas sensor based on polyaniline-multiwalled carbon nanotubes (PANI/MWCNTs) nanocomposite for trace-level ammonia detection. *Sens. Actuators B Chem.* **2015**, *221*, 1523–1534. [CrossRef]
121. Thanasamy, D.; Jesuraj, D.; Avadhanam, V.; Chinnadurai, K.; Kann, S.K.K. Microstructural effect of various polyaniline-carbon nanotube core-shell nanocomposites on electrochemical supercapacitor electrode performance. *J. Energy Storage* **2022**, *53*, 105087. [CrossRef]
122. Jiménez, P.; Castell, P.; Sainz, R.; Ansón, A.; Martínez, M.T.; Benito, A.M.; Maser, W.K. Carbon Nanotube Effect on Polyaniline Morphology in Water Dispersible Composites. *J. Phys. Chem. B* **2010**, *114*, 1579–1585. [CrossRef] [PubMed]
123. Awata, R.; Shehab, M.; El Tahan, A.; Soliman, M.; Ebrahim, S. High performance supercapacitor based on camphor sulfonic acid doped polyaniline/multiwall carbon nanotubes nanocomposite. *Electrochim. Acta* **2020**, *347*, 136229. [CrossRef]
124. Xavier, P.A.F.; Benoy, M.D.; Stephen, S.K.; Varghese, T. Enhanced electrical properties of polyaniline carbon nanotube composites: Analysis of temperature dependence of electrical conductivity using variable range hopping and fluctuation induced tunneling models. *J. Solid State Chem.* **2021**, *300*, 122232. [CrossRef]
125. Asen, P.; Shahrokhian, S.; Zad, A.I. Transition metal ions-doped polyaniline/rapheme oxide nanostructure as high performance electrode for supercapacitor applications. *J. Solid State Electrochem.* **2018**, *22*, 983–996. [CrossRef]
126. Saini, P.; Choudhary, V.; Singh, B.P.; Mathur, R.B.; Dhawan, S.K. Polyaniline–MWCNT nanocomposites for microwave absorption and EMI shielding. *Mater. Chem. Phys.* **2009**, *113*, 919–926. [CrossRef]
127. Ahmed, F.; Kumar, S.; Arshi, N.; Anwar, M.S.; Su-Yeon, L.; Kil, G.-S.; Park, D.-W.; Koo, B.H.; Lee, C.G. Preparation and characterizations of polyaniline (PANI)/ZnO nanocomposites film using solution casting method. *Thin Solid Film.* **2011**, *519*, 8375–8378. [CrossRef]
128. Kocherginsky, N.M.; Lei, W.; Wang, Z. Redox Reactions without Direct Contact of the Reactants. Electron and Ion coupled Transport through Polyaniline Membrane. *J. Phys. Chem. A* **2005**, *109*, 4010–4016. [CrossRef]
129. Nunes, W.G.; Pires, B.M.; Thaines, E.H.N.S.; Pereira, G.M.A.; da Silva, L.M.; Freitas, R.G.; Zanin, H.J. Operando Raman spectroelectrochemical study of polyaniline degradation: A joint experimental and theoretical analysis. *J. Energy Storage* **2022**, *55*, 105770. [CrossRef]
130. Vijayakumar, T.; Joe, I.H.; Nair, C.P.R.; Jayakumar, V.S. Efficient p-electrons delocalization in prospective push–pull non-linear optical chromophore 4-[N,N-dimethylamino]-40-nitro stilbene (DANS): A vibrational spectroscopic study. *Chem. Phys.* **2008**, *343*, 83–99. [CrossRef]
131. Salvatierra, R.V.; Oliveira, M.M.; Zarbin, A.J.G. One-Pot Synthesis and Processing of Transparent, Conducting, and Freestanding Carbon Nanotubes/Polyaniline Composite Films. *Chem. Mater.* **2010**, *22*, 5222–5234. [CrossRef]
132. Correa, A.A.; de Araújo, M.A.; Mascaro, L.H.; Mattoso, L.H.C. In situ polymerized polyaniline films over multi-walled carbon nanotubes coatings for enhanced photoelectrochemical performance. *Polymer* **2024**, *298*, 126869. [CrossRef]

133. Yesilyurt, E.I.; Pionteck, J.; Simon, F.; Voit, B. Fabrication of PANI/MWCNT supercapacitors based on a chitosan binder and aqueous electrolyte for enhanced energy storage. *RSC Appl. Polym.* **2023**, *1*, 97. [CrossRef]
134. Cochet, M.; Louarn, G.; Quillard, S.; Buisson, J.P.; Lefrant, S. Theoretical and experimental vibrational study of emeraldine in salt form. Part II. *J. Raman Spectrosc.* **2000**, *31*, 1041–1049. [CrossRef]
135. Paul, S.J.; Gupta, B.K.; Chandra, P. Probing the electrical and dielectric properties of polyaniline multiwalled carbon nanotubes nanocomposites doped in different protonic acids. *Polym. Bull.* **2021**, *78*, 5667–5683. [CrossRef]
136. Lindfors, T.; Ivaska, A. Raman based pH measurements with polyaniline. *J. Electroanal. Chem.* **2005**, *580*, 320–329. [CrossRef]
137. Acisli, O.; Khataee, A.; Karaca, S.; Sheydae, M. Modification of nanosized natural montmorillonite for ultrasound enhanced adsorption of Acid Red 17. *Ultrason. Sonochemistry* **2016**, *31*, 116–121. [CrossRef]
138. Šeděnková, I.; Trchová, M.; Stejskal, J. Thermal degradation of polyaniline films prepared in solutions of strong and weak acids and in water—FTIR and Raman spectroscopic studies. *Polym. Degrad. Stab.* **2008**, *93*, 2147–2157. [CrossRef]
139. Rozlívková, Z.; Trchová, M.; Šeděnková, I.; Špírková, M.; Stejskal, J. Structure and stability of thin polyaniline films deposited in situ on silicon and gold during precipitation and dispersion polymerization of aniline hydrochloride. *Thin Solid Film.* **2011**, *519*, 5933–5941. [CrossRef]
140. Mahalakshmi, S.; Sridevi, V. Tailoring the synergy between polyaniline and reduced graphene oxide using organic acid dopant, pTSA for enhanced performance as electrode material for supercapacitor applications. *J. Phys. Chem. Solids* **2022**, *165*, 110673. [CrossRef]
141. Abbasi, S.; Hasanpour, M. Variation of the photocatalytic performance of decorated MWCNTs (MWCNTs-ZnO) with pH for photo degradation of methyl orange. *J. Mater. Sci. Mater. Electron.* **2017**, *28*, 11846–11855. [CrossRef]
142. Choudhury, A. Carboxyl-functionalized MWCNT doped poly(o-toluidine) nanohybrids: Synthesis, characterization with AC electrical and dielectric properties. *Synth. Met.* **2014**, *188*, 13–20. [CrossRef]
143. Das, M.; Akbar, A.; Sarkar, D. Investigation on dielectric properties of polyaniline (PANI) sulphonic acid (SA) composites prepared by interfacial polymerization. *Synth. Met.* **2019**, *249*, 69–80. [CrossRef]
144. Zuppiroli, L.; Bussac, M.N.; Paschen, S.; Chauvet, O.; Forro, L. Hopping in disordered conducting polymers. *Phys. Rev.* **1994**, *50*, 5196–5203. [CrossRef]
145. Shi, S.-L.; Zhang, L.-Z.; Li, J.-S. Electrical and dielectric properties of multiwall carbon nanotube/polyaniline composites. *J. Polym. Res.* **2009**, *16*, 395–399. [CrossRef]
146. Abalyaeva, V.V.; Tkachenko, L.I.; Nikolaeva, G.V.; Orlov, A.V.; Kiseleva, S.G.; Efimov, O.N.; Karpacheva, G.P. synthesis and electrochemical properties of nanocomposites containing poly(3,6-bis(phenylamino)-2,5-dichlorobenzoquinone and multiwalled carbon nanotubes. *Polym. Science. Ser. B* **2017**, *59*, 459–471. [CrossRef]
147. Muzaffar, A.; Ahamed, M.B.; Deshmukh, K.; Thirumalai, J. A review on recent advances in hybrid supercapacitors: Design, fabrication and applications. *Renew. Sustain. Energy. Rev.* **2019**, *101*, 123–145. [CrossRef]
148. Zeng, S.; Chen, H.; Cai, F.; Kang, Y.; Chen, M.; Li, Q. Electrochemical fabrication of carbon nanotube/polyaniline hydrogel film for all-solid-state flexible supercapacitor with high areal capacitance. *J. Mater. Chem.* **2015**, *47*, 23864–23870. [CrossRef]
149. Abalyaeva, V.V.; Bogatyrenko, V.R.; Anoshkin, I.V.; Efimov, O.N. Composite Materials Based on Polyaniline and Multiwalled Carbon Nanotubes: Morphology and Electrochemical Behavior. *Polym. Sci. Ser. B* **2010**, *52*, 252–262. [CrossRef]
150. Zhang, J.; Kong, L.-B.; Wang, B.; Luo, Y.-C.; Kang, L. In-situ electrochemical polymerization of multi-walled carbon nanotube/polyaniline composite films for electrochemical supercapacitors. *Synth. Met.* **2009**, *159*, 260–266. [CrossRef]
151. Arami, H.; Mazloumi, M.; Khalifehzadeh, R.; Emami, S.H.; Sadrnezhaad, S.K. Performance of the chemical and Electrochemical Composites of PPy/CNT as Electrodes in Type I Supercapacitors. *J. Nanomater.* **2015**, *2015*, 560164. [CrossRef]
152. Abalyaeva, V.V.; Nikolaeva, G.V.; Efimov, O.N. Preparation and Study of Polyaniline and Multiwalled Carbon Nanotube based Composite Materials. *Russ. J. Electrochem.* **2008**, *44*, 828–834. [CrossRef]
153. Lindfors, T.; Latonen, R.M. Improved charging/discharging behavior of electropolymerized nanostructured composite films of polyaniline and electrochemically reduced graphene oxide. *Carbon* **2014**, *69*, 122–131. [CrossRef]
154. Li, K.; Liu, X.; Chen, S.; Pan, W.; Zhang, J. A flexible solid-state supercapacitor based on graphene/polyaniline paper electrodes. *J. Energy Chem.* **2019**, *32*, 166–173. [CrossRef]
155. Ansari, M.O.; Mohammad, F. Thermal stability and electrical properties of dodecyl-benzene-sulfonic-acid doped nanocomposites of polyaniline and multi-walled carbon nanotubes. *Compos. Part B Eng.* **2012**, *43*, 3541–3548. [CrossRef]
156. Bhadra, S.; Khastgir, D. Glass–rubber transition temperature of polyaniline: Experimental and molecular dynamic simulation. *Synth. Met.* **2009**, *159*, 1141–1146. [CrossRef]
157. Arulmani, S.; Wu, J.J.; Anandan, S. Amphiphilic Triblock Copolymer guided Polyaniline embraced CNT nanohybrid with outcropping whiskers as an energy storage electrode. *Electrochim. Acta* **2017**, *246*, 737–747. [CrossRef]
158. Rahman, M.M.; Shawon, M.R.; Rahman, M.H.; Alam, I.; Faruk, M.O.; Khan, M.M.R.; Okoli, O. Synthesis of polyaniline-graphene oxide based ternary nanocomposite for supercapacitor application. *J. Energy Storage* **2023**, *67*, 107615. [CrossRef]

159. Morávková, Z.; Trchová, M.; Tomsík, E.; Cechvala, J.; Stejskal, J. Enhanced thermal stability of multi-walled carbon nanotubes after coating with polyaniline salt. *Polym. Degrad. Stab.* **2012**, *97*, 1405–1414. [CrossRef]
160. Chen, C.-H. Thermal and Morphological Studies of Chemically Prepared Emeraldine-Base-Form Polyaniline Powder. *J. Appl. Polym. Sci.* **2003**, *89*, 2142–2148. [CrossRef]
161. Bandyopadhyaya, R.; Nativ-Roth, E.; Regev, O.; Yerushalmi-Rozen, R. Stabilization of Individual Carbon Nanotubes in Aqueous Solutions. *Nano Lett.* **2002**, *2*, 25–28. [CrossRef]
162. Strano, M.S.; Moore, V.C.; Miller, M.K.; Allen, M.J.; Haroz, E.H.; Kittrell, C.; Hauge, R.H.; Smalley, R.E. The Role of Surfactant Adsorption during Ultrasonication in the Dispersion of Single-Walled Carbon Nanotubes. *J. Nanosci. Nanotech.* **2003**, *3*, 81–86. [CrossRef]
163. Vargas, L.R.; Poli, A.K.D.S.; Dutra, R.d.C.L.; De Souza, C.B.; Baldan, M.R.; Gonçalves, E.S. Formation of Composite Polyaniline and Graphene Oxide by Physical Mixture Method. *J. Aerosp. Technol. Manag.* **2017**, *9*, 29–38. [CrossRef]

Disclaimer/Publisher's Note: The statements, opinions and data contained in all publications are solely those of the individual author(s) and contributor(s) and not of MDPI and/or the editor(s). MDPI and/or the editor(s) disclaim responsibility for any injury to people or property resulting from any ideas, methods, instructions or products referred to in the content.

https://doi.org/10.3390/polym18060000
